# Age-specific contribution of contacts to transmission of SARS-CoV-2 in Germany

**DOI:** 10.1007/s10654-022-00938-6

**Published:** 2023-01-03

**Authors:** Isti Rodiah, Patrizio Vanella, Alexander Kuhlmann, Veronika K. Jaeger, Manuela Harries, Gerard Krause, Andre Karch, Wolfgang Bock, Berit Lange

**Affiliations:** 1 Department of Epidemiology, Helmholtz Centre for Infection Research (HZI), Inhoffenstr. 7, DE-38124 Brunswick, Germany; 2 Chair of Empirical Methods in Social Science and Demography, University of Rostock, Ulmenstr. 69, DE-18057 Rostock, Germany; 3 Faculty of Medicine, Martin Luther University Halle-Wittenberg, Magdeburgerstr. 8, DE-06112 Halle (Saale), Germany; 4 German Center for Lung Research (DZL), Biomedical Research in End-Stage and Obstructive Lung Disease Hannover (BREATH), Carl-Neuberg-Str. 1, DE-30625 Hannover, Germany; 5 Institute of Epidemiology and Social Medicine, University of Münster, Albert-Schweitzer-Campus 1, DE-48149 Münster, Germany; 6 Technomathematics Group, Department of Mathematics, TU Kaiserslautern, Gottlieb-Daimler-Straße 48, DE-67663 Kaiserslautern, Germany; 7 German Centre for Infection Research (DZIF), Inhoffenstr. 7, DE-38124 Brunswick, Germany

**Keywords:** Epidemiological modeling, Compartment models, Age-specific viral spread, Underdetection, Infection severity, SARS-CoV-2, COVID-19

## Abstract

Current estimates of pandemic SARS-CoV-2 spread in Germany using infectious disease models often do not use age-specific infection parameters and are not always based on age-specific contact matrices of the population. They also do usually not include setting- or pandemic phase-based information from epidemiological studies of reported cases and do not account for age-specific underdetection of reported cases. Here, we report likely pandemic spread using an age-structured model to understand the age- and setting-specific contribution of contacts to transmission during different phases of the COVID-19 pandemic in Germany. We developed a deterministic SEIRS model using a pre-pandemic contact matrix. The model was optimized to fit age-specific SARS-CoV-2 incidences reported by the German National Public Health Institute (Robert Koch Institute), includes information on setting-specific reported cases in schools and integrates age- and pandemic period-specific parameters for underdetection of reported cases deduced from a large population-based seroprevalence studies. Taking age-specific underreporting into account, younger adults and teenagers were identified in the modeling study as relevant contributors to infections during the first three pandemic waves in Germany. For the fifth wave, the Delta to Omicron transition, only age-specific parametrization reproduces the observed relative and absolute increase in pediatric hospitalizations in Germany. Taking into account age-specific underdetection did not change considerably how much contacts in schools contributed to the total burden of infection in the population (up to 12% with open schools under hygiene measures in the third wave). Accounting for the pandemic phase and age-specific underreporting is important to correctly identify those groups of the population in which quarantine, testing, vaccination, and contact-reduction measures are likely to be most effective and efficient. Age-specific parametrization is also highly relevant to generate informative age-specific output for decision makers and resource planers.

**Supplementary Information:**

The online version contains supplementary material available at 10.1007/s10654-022-00938-6.

## Introduction

Epidemiological models have been essential to predict the spread of SARS-CoV-2 and are frequently used to inform decision-makers about the effectiveness of interventions. Many approaches for projection estimations and scenario modeling use compartment models that divide a population by health status and assume transition rates from one health status to another [2]. Simulations are based on assumptions or data about the probability of disease transmission, incubation period, recovery rates, and mortality rates. Based on deterministic differential equations, multiple studies have modeled the current spread of SARS-CoV-2 (e.g. [11, 24]). Some models consider age groups [24, 26, 34, 36], are agent-based [44], or include mobility [35]. Others consider additional disease compartments [24] and vaccination [36]. However, current models for Germany are not sufficiently accounting for age-specific estimates of disease severity and underdetection of reported cases, which leads to underestimation as well as overestimation of the contribution of contacts in different age and population groups to infection dynamics and deaths [47]. Even during the fifth SARS-CoV-2 wave in Germany (Delta to Omicron transition), models informing policy makers were not age-specific [29], although disease severity as well as vaccination coverage [51] was clearly age-dependent [52, 54].

The population’s age structure is known as one of the key determinants of acute respiratory diseases, especially when it comes to infection severity. For instance, children are considered to be responsible for a large part of the transmission of influenza [4] but the majority of hospitalizations and deaths occur among people of ages over 65 years [31, 40]. Similarly, COVID-19 mortality among people who have been tested positive for SARS-CoV-2 is substantially higher in older age groups and very low for young children [33, 43, 45]. Moreover, the infectiousness of an individual has been reported to vary with time after infection dependent on age of that individual [14, 18], which is known to affect epidemic spread [20, 23]. As for the modeling of all respiratory virus infections, age-specific contact patterns in the population are additionally relevant, as they define epidemic patterns of transmission [28, 30, 32].

For early variants of SARS-CoV-2 [46], there was preliminary evidence that children had lower susceptibility to SARS-CoV-2 infection than adults, with adolescents appearing to have a similar susceptibility to adults. In pooled estimates from worldwide population-based studies even early on in the pandemic [25], estimated age-specific infection fatality ratio was very low for children and younger adults but increased progressively with increasing age. Hence, age-stratified transmission models with heterogeneous contact rates between age groups have been e.g. developed and fitted to the COVID-19 epidemic data from China, Italy, Japan, Singapore, Canada, and South Korea [7]. In a similar way, a modeling framework was developed to reconstruct the complex patterns of SARS-CoV-2 spread across age groups along with the dynamics of infections and hospitalizations in France [41].

Underdetection of actual infection activity by notified case reports to authorities is a well-known limitation, but not often included in modeling efforts, even less using age-specific underreporting estimates [24, 35]. For Germany, population-based studies suggest that actual infection activity is heterogeneous across regions, time phases, and age groups [15]. However, both age and underdetection of infections are highly relevant for predicting regional infection events, for estimating the effectiveness of interventions [17] as well as for predicting hospitalizations and deaths correctly.

In the work presented here, we incorporate age-specific underdetection ratios in a classical age-specific infection model and analyze the impact on transmission dynamics. The model has a circular structure, which allows for reinfections, long-term complications, and delayed deaths. Moreover, we consider age-specific social contact patterns since this impacts the spread of disease. With this, our model allows us to assess the age-dependent contribution of contacts to the transmission of SARS-CoV-2 in Germany, both when taking underdetection ratios into account and when not. We assess the sensitivity of infection transmission by applying underdetection ratios from large population-based seroprevalence studies of adults [15] and children [21, 42]. We then analyze and compare the transmission dynamics across age groups during the first three infection waves in Germany, taking underdetection into account. We also estimate time-dependent transmission rates and the contribution of contacts within the school setting for periods where specific data on schools are available [55]. Moreover, we give an example of how age-specific models are essential in providing relevant and informative output to decision makers, by assessing the SARS-CoV-2vDelta-Omicron transition (the fifth wave) in Germany.

## Data and methods

### Data

**Fig. 1:**
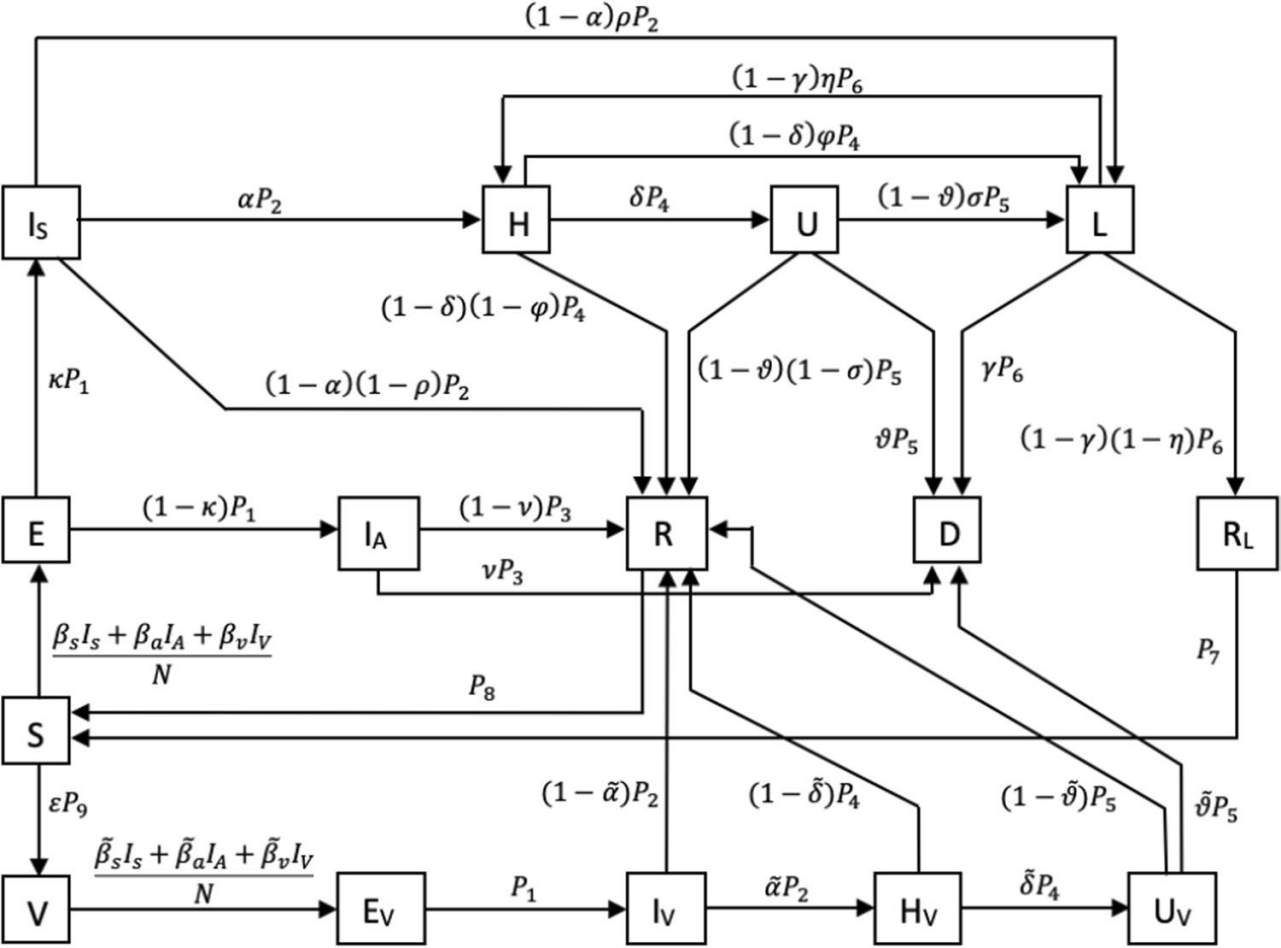
Schematic illustration of the extended SEIRS model

We considered data on officially reported SARS-CoV-2 infections by the German National Public Health Institute (Robert Koch Institute; RKI) [53]. The age distribution of the German population is obtained from data for 2020 reported by the Federal Statistical Office [49]. We used weekly reported age-specific data of SARS-CoV-2 infections during the first three infection waves in Germany, i.e. calendar week 5 (starting on 27 January) 2020 to calendar week (starting on 23 June) 20 of 2021. Weekly reported SARS-CoV-2 infections by age group are shown in Supplementary A–Fig. 1.

**Fig. 2:**
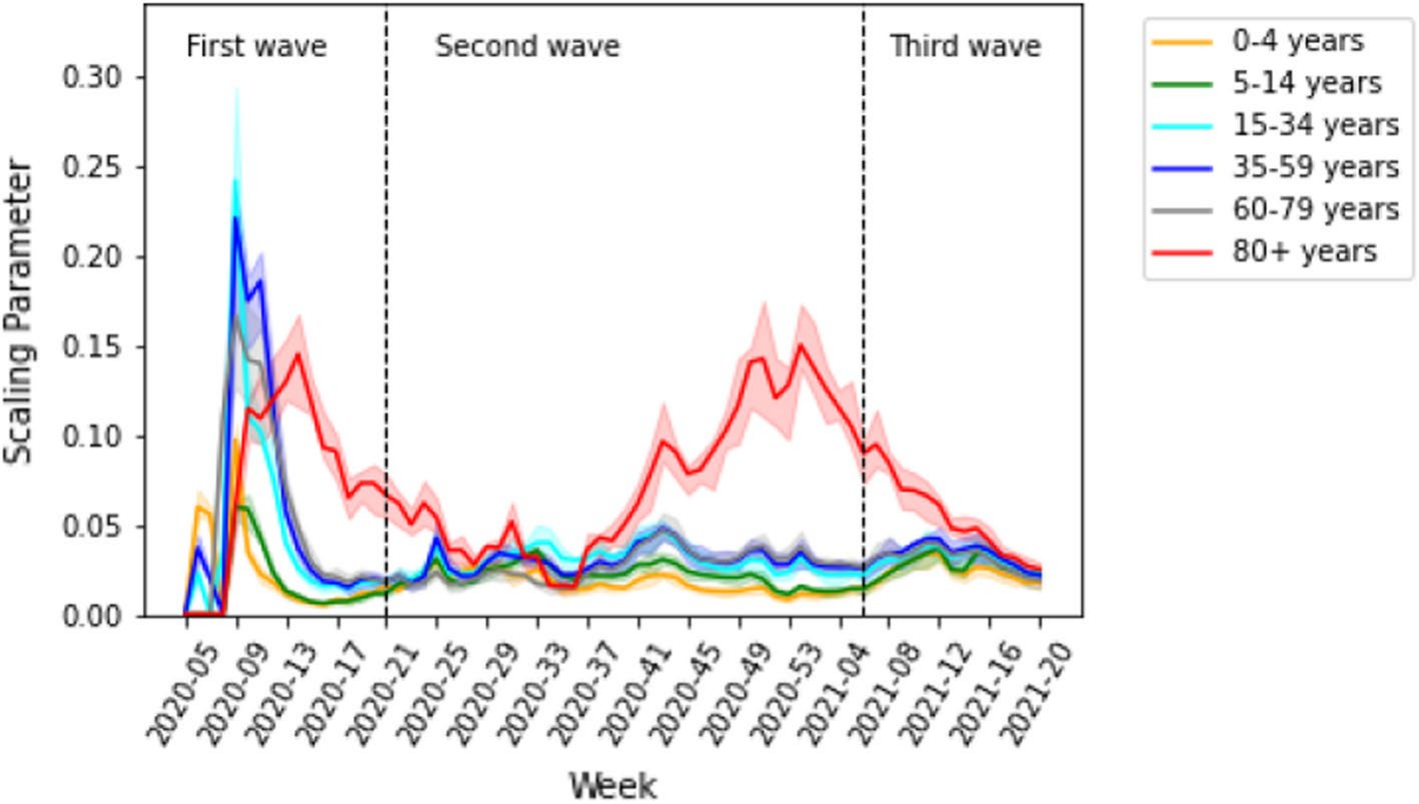
Estimated weekly scaling parameters (CI: 95%) per contact by age group without accounting for underdetection

To investigate the contribution of school contacts, we use the weekly reported SARS-CoV-2 infections among students and teachers from the Standing Conference of Ministers of Education and Cultural Affairs website (KMK) [55]. The data used are from calendar week 8 to 30 of 2021 (Supplementary A–Fig. 2). We assumed that the proportion of new infections in schools for different age groups is determined by the proportion of student and teacher numbers reported by the Federal Ministry of Education and Research [48] and the Federal Statistical Office [50].

Based on pandemic phase- and age-specific underdetection ratios derived from population-based studies among adults [15] and children [21, 22, 42], we estimated average age-specific underdetection ratios for different phases of the pandemic in Germany (see Table 1), which we implemented in our model. Gornyk et al. [15] investigated seroprevalence estimates for SARS-CoV-2 that indicated an age-specific underreporting ratio (infected to reported) of SARS-CoV-2 transmission in a large (> 25,000 participants, seven large regions in Germany) population-based seroprevalence study and we used their estimates for age-specific underdetection ratios in adults. For children, we used seroprevalence studies available for the south of Germany during the first and second waves [21, 22, 42]. For the third wave, we assumed a significantly reduced level of underdetection in this age group, based on reported cases during the period of the introduction of large-scale testing in child care institutions, as no seroprevalence estimates for children were available. To apply underdetection in our model, we correlated the age-specific underdetection ratios of each stage to the reported number of infected individuals.

**Table 1:** Underdetection ratios by age group for different SARS-CoV-2 waves of transmission (Source: [13, 18, 19, 35])

Group	First wave (March–June 2020)	Second wave (July 2020–February 2021)	Third wave (March–May 2021)
0–4 years	8	6	2
5–14 years	6	4	1.5
15–34 years	6	4	2
35–59 years	4	3	2
60–79 years	4	4	3
80 + years	2	1.5	1.5

**Fig. 3:**
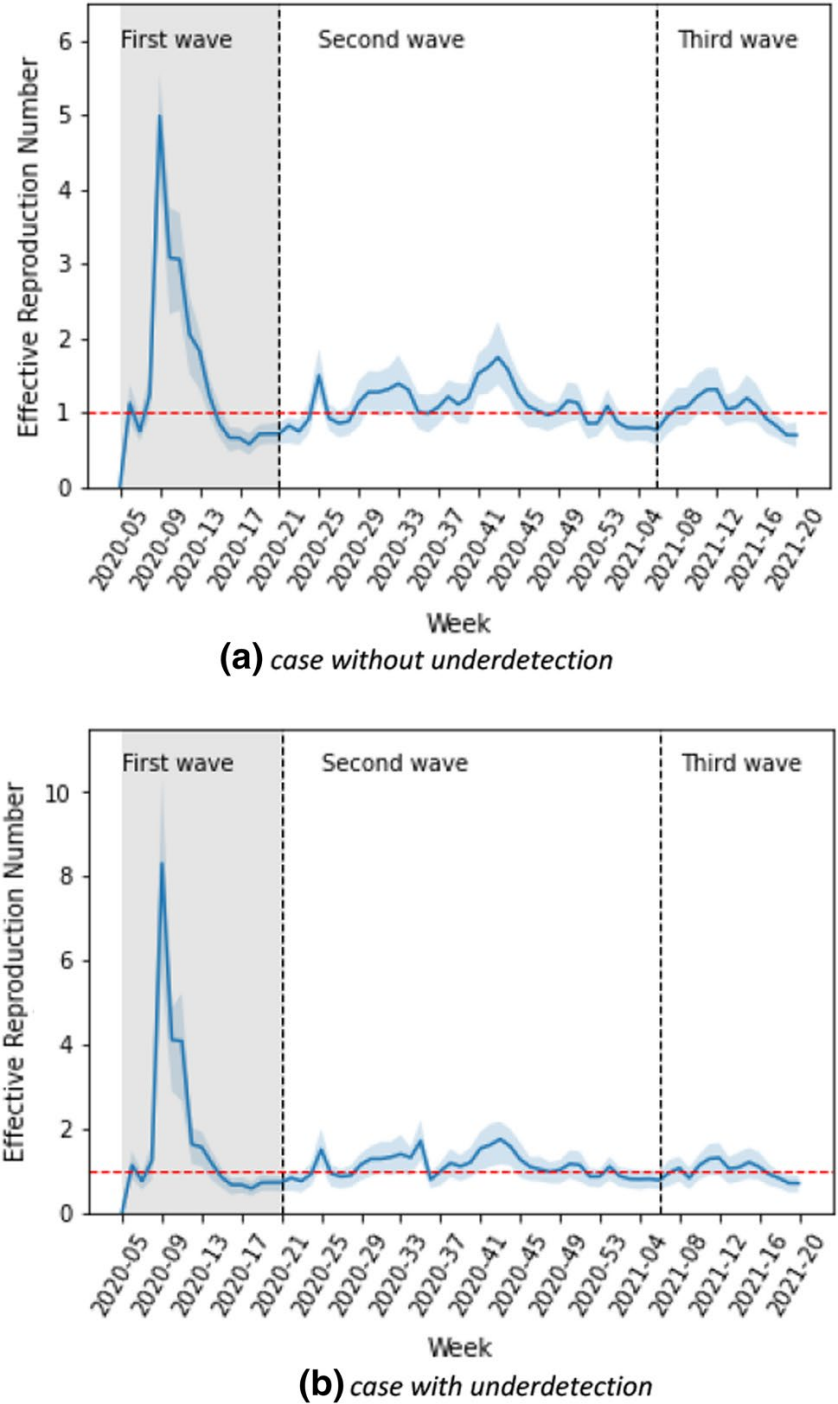
The effective reproduction number is calculated by fitting parameters for case **a** without and **b** with underdetection In the first few weeks of the first wave, there were artifacts due to a low number of cases, increased testing, etc. Therefore, the results of weekly calibration are unrealizable during the first few weeks

Since our age-structured model allows us to adjust the transmission rates among different age groups, we applied social contact patterns to the transmission rates. We used POLYMOD data for Germany, originally made available by Mossong et al. [32], to construct a symmetric contact rate matrix by age group consistent with our model. The contact rate ${c}_{ij}$ is related to the social contact matrix by

1 $${c}_{ij}=\frac{{m}_{ji}}{{N}_{i}},$$

where the element ${m}_{ij}$ of the social contact matrix (see Supplementary A–Fig. 3) represents the average number of contacts made by an individual in the age group $i$ with individuals in the age group $j$ during one day and ${N}_{i}$ is the population size of age group $i$.

### Model

The developed model is a deterministic SEIRS (Susceptible-Exposed-Infectious-Recovered-Susceptible) model adapted to the specific properties of SARS-CoV-2 infections. It distinguishes healthy (susceptible) individuals, infected but not yet infectious (exposed) individuals, symptomatic and asymptomatic patients. Furthermore, we considered compartments for hospitalizations, patients in the intensive care units (ICUs), and long-COVID (i.e. suffering eventual symptoms after officially recovering from the infection). Last, patients will recover or die. We also assumed that there is a reinfection process. In our model, we split the recovery compartment into a compartment for those recovered from COVID and a long-COVID compartment, since we assumed that both have a different reinfection probability. Since the start of vaccination in early 2021 in Germany, we consider vaccination compartments for individuals who were second vaccinated. For this we included compartments for exposed, infectious, hospitalizations, patients in the intensive care units (ICUs) for individuals after vaccination, as we assume that vaccination could reduce the risk of infection, severe disease, and death. Figure 1 illustrates the model structure.

The population is split into six age groups, as given in Table 2, in accordance with data provided by RKI. An individual in age group $i$ is classified either as susceptible (${S}_{i}$), exposed (${E}_{i}$), asymptomatically infectious (${{I}_{A}}_{i}$), symptomatically infectious (${{I}_{S}}_{i}$), hospitalized (${H}_{i}$), in intensive care (${U}_{i}$), suffering under long-COVID (${L}_{i}$), fully recovered (${{R}_{F}}_{i}$), recovered from long-COVID (${{R}_{L}}_{i}$), or dead (${D}_{i}$). Since the start of vaccination in early 2021, an individual in the age group over 60 years can also be classified as either vaccinated (${V}_{i}$), exposed after vaccination (${{E}_{V}}_{i}$), infectious after vaccination (${{I}_{V}}_{i}$), hospitalized after vaccination (${{H}_{V}}_{i}$), or in intensive care after vaccination (${{U}_{V}}_{i}$). The population size of age group $i$ is given by ${N}_{i}$. The developed model is given by the following differential equation system,

2 $$\begin{gathered} \frac{{dS_{i} \left( t \right)}}{dt} = - \mathop \sum \limits_{j} \Lambda_{ij} \left( t \right)S_{i} \left( t \right) + P_{7} R_{Li} \left( t \right) + P_{8} R_{Fi} \left( t \right) - \varepsilon P_{9} S_{i} \left( t \right), \hfill \\ \frac{{dV_{i} \left( t \right)}}{dt} = - \mathop \sum \limits_{j} \tilde{\Lambda }_{ij} \left( t \right)V_{i} \left( t \right) + \varepsilon P_{9} S_{i} \left( t \right), \hfill \\ \frac{{dE_{i} \left( t \right)}}{dt} = \mathop \sum \limits_{j} \Lambda_{ij} \left( t \right)S_{i} \left( t \right) - P_{1} E_{i} \left( t \right), \hfill \\ \frac{{dE_{Vi} \left( t \right)}}{dt} = \mathop \sum \limits_{j} \tilde{\Lambda }_{ij} \left( t \right)V_{i} \left( t \right) - P_{1} E_{Vi} \left( t \right), \hfill \\ \frac{{dI_{Si} \left( t \right)}}{dt} = \kappa_{i} P_{1} E_{i} \left( t \right) - P_{2} I_{Si} \left( t \right), \hfill \\ \frac{{dI_{Ai} \left( t \right)}}{dt} = \left( {1 - \kappa_{i} } \right)P_{1} E_{i} \left( t \right) - P_{3} I_{Ai} \left( t \right), \hfill \\ \frac{{dI_{Vi} \left( t \right)}}{dt} = P_{1} E_{Vi} \left( t \right) - P_{2} I_{Vi} \left( t \right), \hfill \\ \frac{{dH_{i} \left( t \right)}}{dt} = \alpha_{i} P_{2} I_{Si} \left( t \right) - P_{4} H_{i} \left( t \right) + \left( {1 - \gamma_{i} } \right)\eta_{i} P_{6} L_{i} \left( t \right), \hfill \\ \frac{{dH_{Vi} \left( t \right)}}{dt} = \tilde{\alpha }_{i} P_{2} I_{Vi} \left( t \right) - P_{4} H_{Vi} \left( t \right), \hfill \\ \frac{{dU_{i} \left( t \right)}}{dt} = \delta_{i} P_{4} H_{i} \left( t \right) - P_{5} U_{i} \left( t \right), \hfill \\ \frac{{dU_{Vi} \left( t \right)}}{dt} = \tilde{\delta }_{i} P_{4} H_{Vi} \left( t \right) - P_{5} U_{Vi} \left( t \right), \hfill \\ \frac{{dL_{i} \left( t \right)}}{dt} = \left( {1 - \alpha_{i} } \right)\rho_{i} P_{2} I_{Si} \left( t \right) + \left( {1 - \delta_{i} } \right)\varphi_{i} P_{4} H_{i} \left( t \right) + \left( {1 - \vartheta_{i} } \right)\sigma_{i} P_{5} U_{i} \left( t \right) - P_{6} L_{i} \left( t \right), \hfill \\ \frac{{dR_{Fi} \left( t \right)}}{dt} = \left( {1 - \nu_{i} } \right)P_{3} I_{Ai} \left( t \right) + \left( {1 - \alpha_{i} } \right)\left( {1 - \rho_{i} } \right)P_{2} I_{Si} \left( t \right) + \left( {1 - \delta_{i} } \right)\left( {1 - \varphi_{i} } \right)P_{4} H_{i} \left( t \right) + \left( {1 - \vartheta_{i} } \right)\left( {1 - \sigma_{i} } \right)P_{5} U_{i} \left( t \right) + \left( {1 - \tilde{\alpha }_{i} } \right)P_{2} I_{Vi} \left( t \right) + \left( {1 - \tilde{\delta }_{i} } \right)P_{4} H_{Vi} \left( t \right) + \left( {1 - \tilde{\vartheta }_{i} } \right)P_{5} U_{Vi} \left( t \right) - P_{8} R_{Fi} \left( t \right), \hfill \\ \frac{{dR_{Li} \left( t \right)}}{dt} = \left( {1 - \gamma_{i} } \right)\left( {1 - \eta_{i} } \right)P_{6} L_{i} \left( t \right) - P_{7} R_{Li} \left( t \right), \hfill \\ \frac{{dD_{i} \left( t \right)}}{dt} = \vartheta_{i} P_{5} U_{i} \left( t \right) + \nu_{i} P_{3} I_{Ai} \left( t \right) + \gamma_{i} P_{6} L_{i} \left( t \right) + \tilde{\vartheta }_{i} P_{5} U_{Vi} \left( t \right), \hfill \\ \end{gathered}$$

$${N}_{i}={S}_{i}+{V}_{i}+{E}_{i}+{{E}_{V}}_{i}+{{I}_{S}}_{i}+{{I}_{A}}_{i}+{{{I}_{V}}_{i}+H}_{i}+{{H}_{V}}_{i}+{U}_{i}+{{U}_{V}}_{i}+{L}_{i}+{{R}_{F}}_{i}+{{R}_{L}}_{i}+{D}_{i},$$

where

**Table 2:** Age groups in our model

$i$	Group
1	0–4 years
2	5–14 years
3	15–34 years
4	35–59 years
5	60–79 years
6	80 + years

3 $${\Lambda }_{ij}\left(t\right)={c}_{ij}\left({b}_{{s}_{i}}\left(t\right){{I}_{S}}_{j}\left(t\right)+{b}_{{a}_{i}}\left(t\right){{I}_{A}}_{j}\left(t\right)+{b}_{{v}_{i}}\left(t\right){{I}_{V}}_{j}\left(t\right)\right),$$

Typically, ${b}_{{s}_{i}}(t)$, ${b}_{{a}_{i}}(t)$, and ${b}_{{v}_{i}}\left(t\right)$ denote the time-dependent risk of infection per contact in age group $i$ for symptomatically, asymptomatically, and vaccinated infectious individuals, respectively. In our model, we call them scaling parameters since we calibrated them to scale the contact matrix to account for the effects of interventions and behavioral change over time. ${P}_{1}-{P}_{9}$ are health state transition rates. The Greek letters denote the transition probabilities. Supplementary B gives an overview of the model parameters.

The basic reproduction number ${R}_{0}$ is defined as the expected number of secondary infections produced by a single infected individual in a completely susceptible population [8], which is used to describe the transmissibility of infectious agents [10]. ${R}_{0}$ can be derived by employing the next generation matrix method [9]. The compartments with infected individuals are divided into two categories, which are the appearance of new infections in the compartment and the transfer of the infected into and out of the compartment. The Jacobian [9] of the transmission matrix $F$ and transition matrix $V$ describes the generation of new infections and the transfer across compartments. The elements ${m}_{ij}$ of $M=F{V}^{-1}$ are related to the expected number of secondary infections in compartment $i$ caused by an infected individual of compartment $j$. During an epidemic, susceptible individuals gradually become infected. Therefore, the effective reproduction number is defined as a time-varying variable that quantifies the instantaneous transmissibility of infections. It also includes the effects of interventions and behavioral changes. The effective reproduction number ${R}_{t}$ for the age-structured model is the dominant eigenvalue of the matrix $M$ given by

4 $${M}_{ij}\left(t\right)={S}_{i}\left(t\right){c}_{ij}\left(\frac{{{b}_{s}}_{i}\left(t\right){\kappa }_{j}}{{P}_{3}}+\frac{{{b}_{a}}_{i}\left(t\right)\left(1-{\kappa }_{j}\right)}{{P}_{2}}\right).$$

Here, ${R}_{t}$ is estimated numerically for a set of parameters and a susceptible population.

### Parameter estimation

The model parameters are fit by ordinary least squares (OLS). The time-dependent scaling parameters (${b}_{{s}_{i}}(t)$ and ${b}_{{a}_{i}}(t)$) are estimated by calibrating them weekly to age-specific reported new SARS-CoV-2 infections. Based on the fitting results, we observe the estimated force of infection corresponding to the age groups. The time-dependent marginal force of infection in age group $i$ with respect to contacts with age group $j$ is estimated by matrices obtained from Eq. (3). The force of infection by age group is the row sum of matrices (3) over age group $j$. We also estimate the effective reproduction number over time by calculating Eq. (4) using the estimated scaling parameters.

We use the general bootstrap method [13] to describe a parametric bootstrapping approach for quantifying parameter uncertainty and establishing confidence intervals in mathematical modeling studies, e.g. [6]. In this method, repeated samples are drawn from the best-fit model to quantify parameter uncertainty, assuming that the time series follows a Poisson distribution based on the mean at the time points of the model to the data.

Inserting the fitted parameters into the model, we predict the number of new cases by age group. In addition, we use the same method to fit the fatality ratio and predict the number of deaths. To evaluate the model, we use the weighted interval score (WIS) by Bracher et al. [5], which is one of the evaluation parameters used in the European COVID-19 Forecast Hub [47]. We also refer to a collaborative work where this is explained further [39].

### Estimation of the age-dependent contribution

By applying social contact patterns and the time-dependent scaling parameters among the different age groups into our model, we estimate the number of new cases in the age group $i$ generated by each contact in the age group $j$ over time through matrices obtained by multiplying Eq. (3) with the number of susceptibles by age group,

5 $${\Lambda }_{ij}\left(t\right)\left({S}_{i}\left(t\right)\cdot {1}_{j}\right),$$

where ${1}_{j}$ denotes a row vector of ones. This allows us to investigate among whom SARS-CoV-2 spreads over time. The results show the contribution of contacts to transmission in the overall population. We divided the observation time into three phases representing the first three waves of infection in Germany. Therefore, we are able to analyze the influence of age-specific contacts in the infection dynamics of individuals of different age groups at the national level from a temporal perspective.

### Implementation of the school setting

For the implementation of the school setting, we developed a metapopulation model with two sub-populations, “school” and “non-school”. In the model, we used two types of social contact patterns, that is a global contact rate, including school contacts, and non-school contact rate. The structure of the model for school-setting can be found in Supplementary D. We divide each sub-population into nine age groups, as given in Table 3.

**Table 3:** Age groups in model of school setting

$\mathrm{i}$	Group
1	0–4 years
2	5–9 years
3	10–14 years
4	15–19 years
5	20–24 years
6	25–29 years
7	30–59 years
8	60–79 years
9	80 + years

We assumed that the age groups 2–8 have direct relevance in the school context (as students and teaching staff). Using OLS, we calibrated our model with the weekly reported SARS-CoV-2 cases among students and teachers in schools. The estimated forces of infection in schools are calculated via Eq. (5) using the school contact matrix by POLYMOD and the number of infections in the school.

### Implementation of underdetection of infections by reported cases

We estimated the true new infections by multiplying the weekly age-specific reported cases with the age-specific underdetection ratios from Table 1,

6 $${TI}_{i}={RI}_{i} \times {r}_{i},$$

where ${r}_{i}$ is the underdetection ratio of age group $i$, and ${TI}_{i}$ and ${RI}_{i}$ denote the estimated true new and reported infected of age group $i$, respectively. Then, we used the same method for fitting data to estimate the time-dependent transmission parameters among different age groups. We investigated the contribution of contacts to transmission, accounting for the underdetection in the overall population. Thus, we can analyze the sensitivity of infection transmission by applying underdetection ratios for Germany.

## Results

### Age-dependent contribution of contacts to cases without accounting for underdetection

Figure 2 shows the time-dependent scaling parameters by age group derived from our model over calendar week 5 of 2020 to calendar week 20 of 2021 in Germany. During the first wave, the scaling parameters were highest for all age groups when the spread of infection began. In the second and third waves, the scaling parameters remained stationary for each age group, except for the elderly age group of 80 years and above. The scaling parameter for the elderly increased in the second wave and then decreased in the third wave. Figure 3a shows the effective reproduction number ${R}_{t}$, calculated by fitting parameters according to the age groups. The value of ${R}_{t}$ was estimated to be around 6.9 in calendar week 9 of 2020 when the spread of infection began in the first wave. At the beginning of calendar week 12 of 2020, ${R}_{t}$ decreased below 2. The ${R}_{t}$ was estimated to be in the range of 0.7–2 in the second wave and between 0.6 and 1.4 in the third wave.

**Fig. 4:**
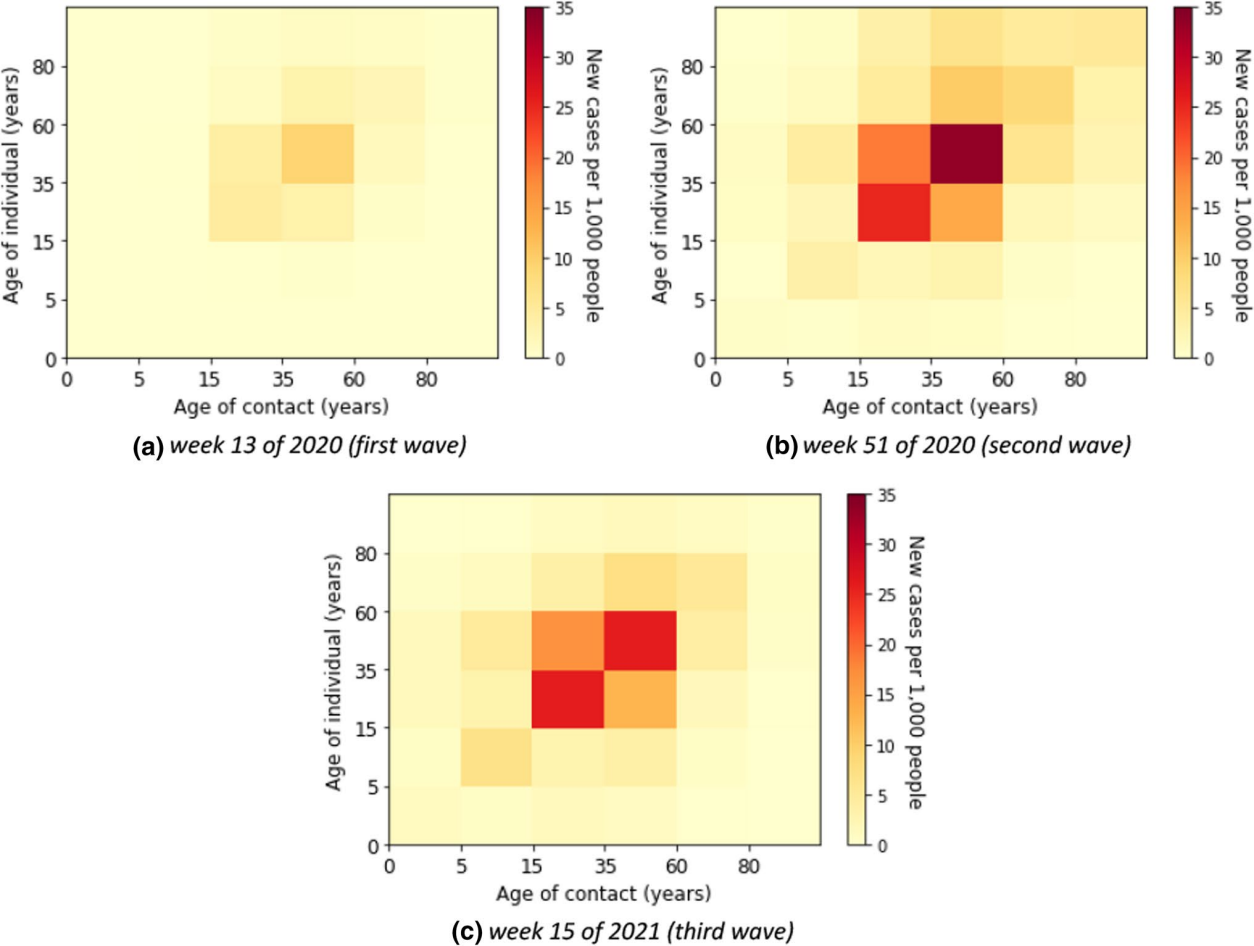
Estimated absolute contribution of contacts to the transmission

Here, we describe the estimated marginal force of infection, which is the individual contribution of contacts to the age-specific transmission rate. More detailed results are shown in heat maps in Supplementary E. The element of a matrix represents an estimator for the force that an individual in the age group on the vertical axis will become infected from individuals in the age group on the horizontal axis. We also show the estimated contributions of contacts to the transmission over time in heat maps in Supplementary F. Some contributions of contacts for the transmission at a fixed time for all waves are shown in Fig. 4. The element of a matrix represents an estimated number of infected in the age group on the vertical axis due to contact with individuals in the age group on the horizontal axis. For instance, in Fig. 4b, we estimated that there were about 18,800 new infections aged 35–59 years due to contacts with individuals aged 15–35 years in week 51 of 2020.

### First wave

**Fig. 5:**
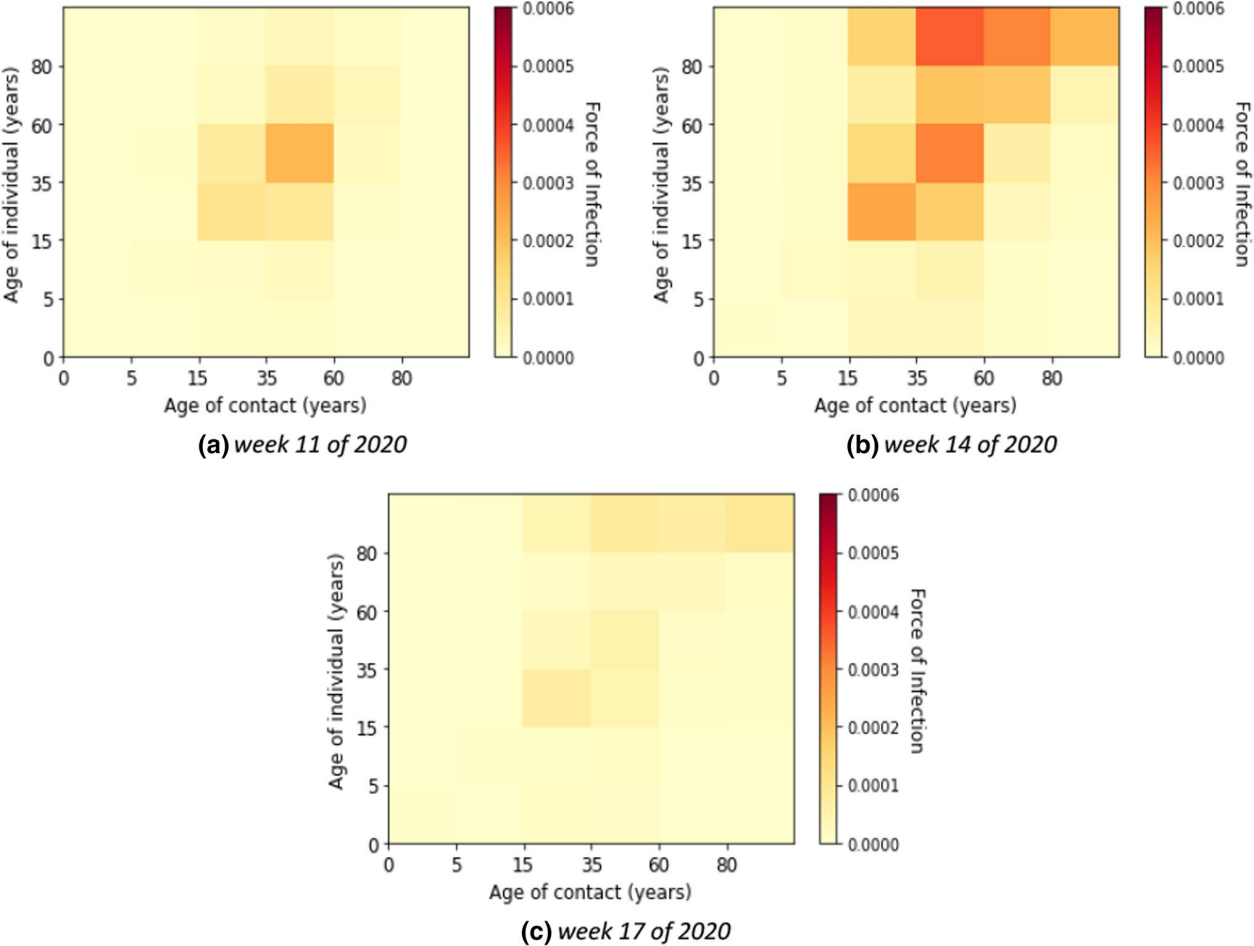
Estimated force of infection in contribution of contacts by age group in the first wave

**Fig. 6:**
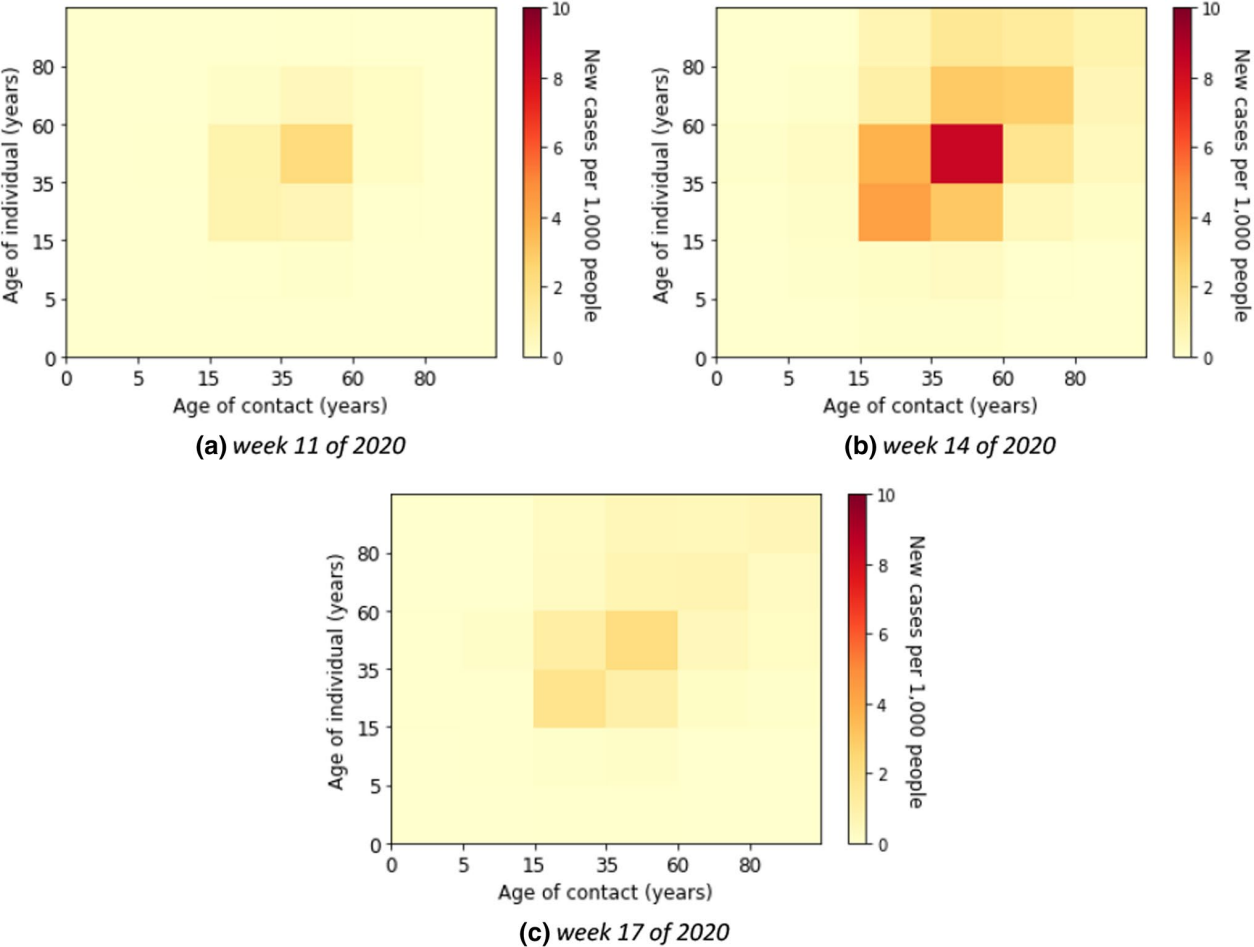
Estimated contribution of contact by age group to transmission in the first wave

Without accounting for underestimation of cases the individual contribution of contacts to the overall transmission rates was predominantly from contacts in young adults, adults, and the elderly (see Fig. 5) in the first wave, i.e. weeks 5 until 21 of 2020. The absolute contribution of contacts to the transmission was highest in the adult group (see Fig. 6 for example of calendar weeks 11, 14, and 17 of 2020 in the first wave and Supplementary F–Fig. 11 for all calendar weeks).

### Second wave

**Fig. 7:**
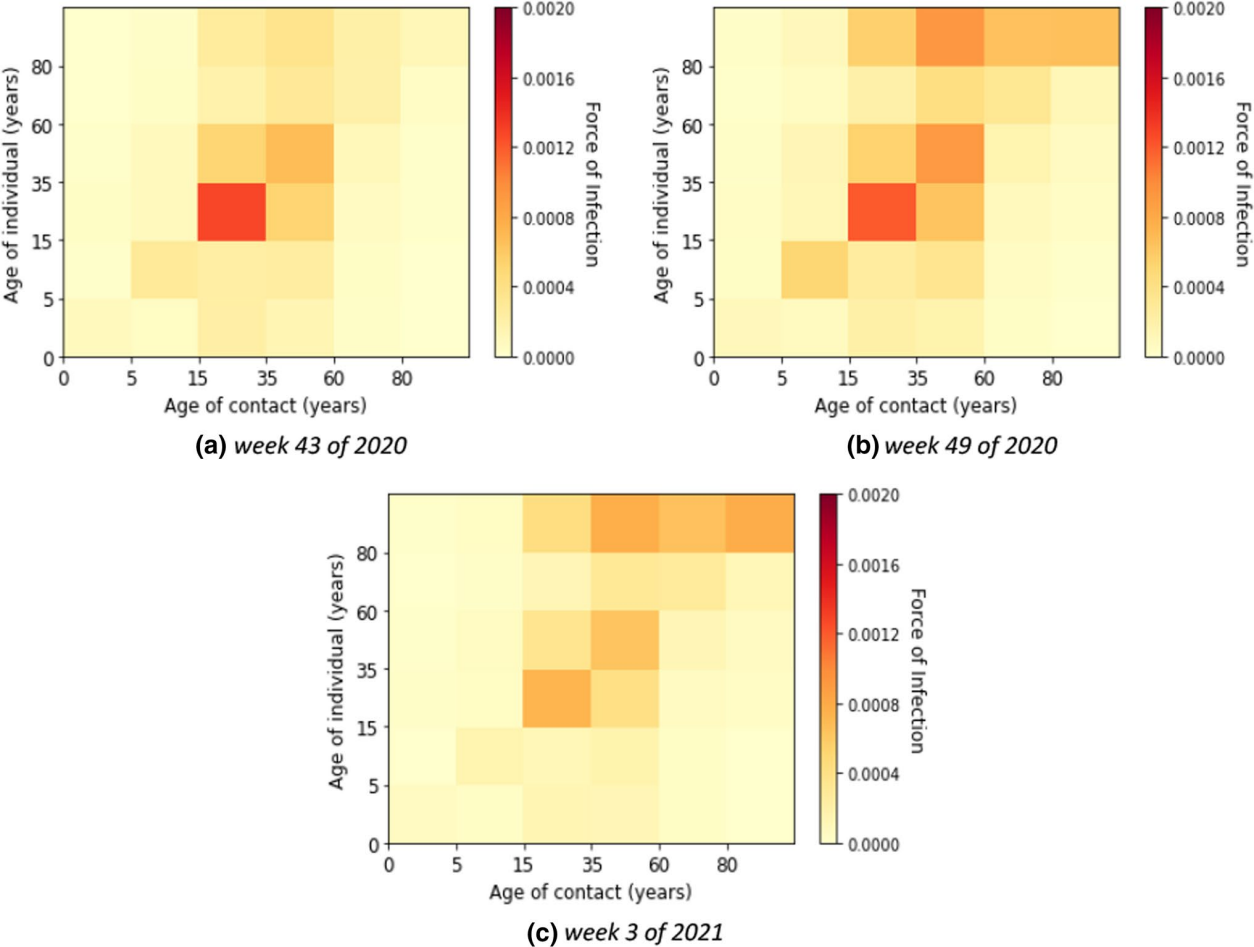
Estimated force of infection in contribution of contacts by age group in the second wave

**Fig. 8:**
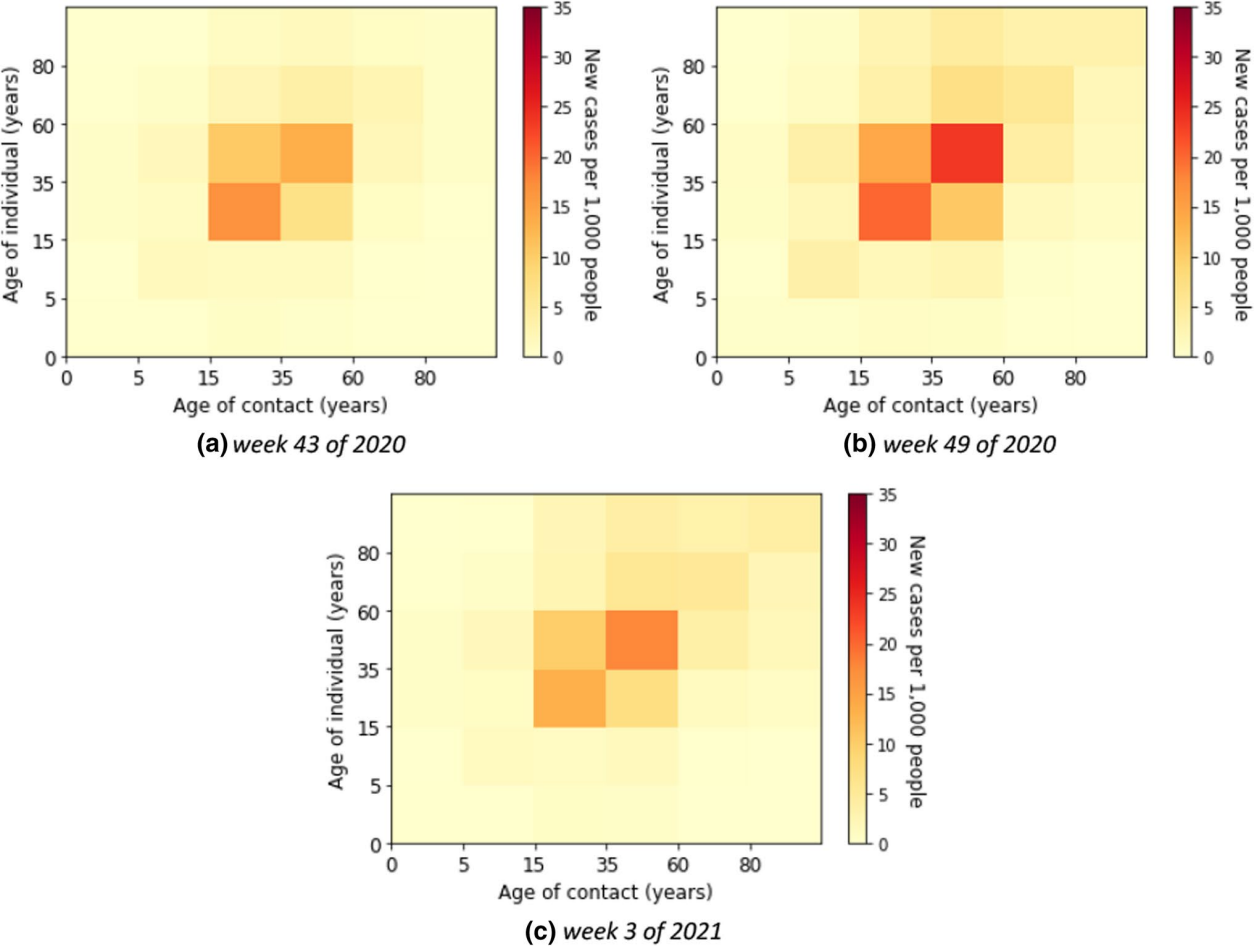
Estimated contribution of contact by age group to transmission in the second wave

In the second wave, the reported number of cases was relevantly higher than in the first wave. The age-specific forces of infection are shown in Fig. 7 for example calendar weeks in the second. The individual contribution of contacts to the transmission rates for the second wave was highest in age groups from young adults to elderly groups. In weeks 50 of 2020 until 2 of 2021, the contribution to the transmission was highest in the elderly that had contact with the other age groups above 14 years. In Fig. 8, we show that the absolute contribution of contacts to the transmission was mainly in the young adult and adult groups.

### Third wave

**Fig. 9:**
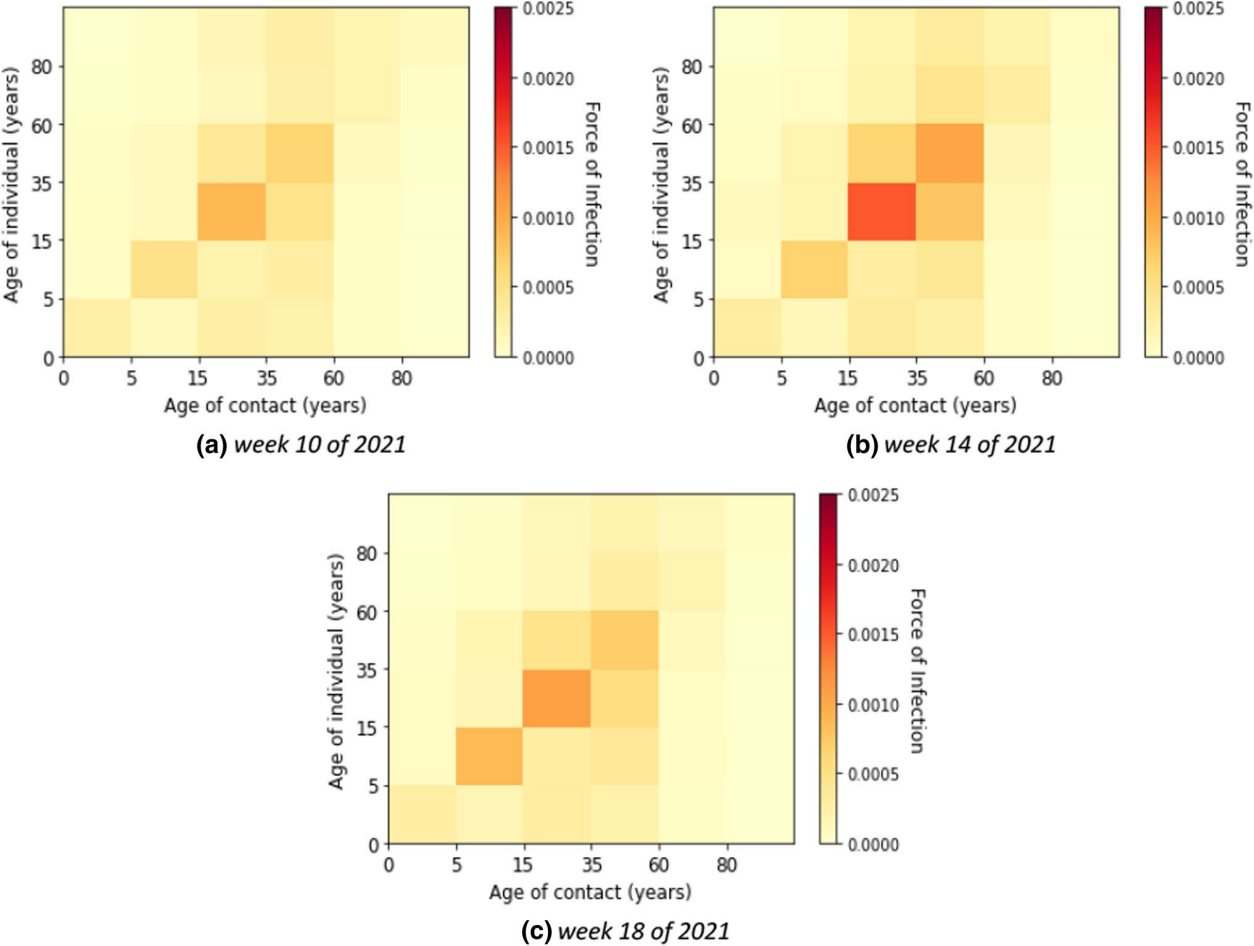
Estimated force of infection in contribution of contacts by age group in the third wave

**Fig. 10:**
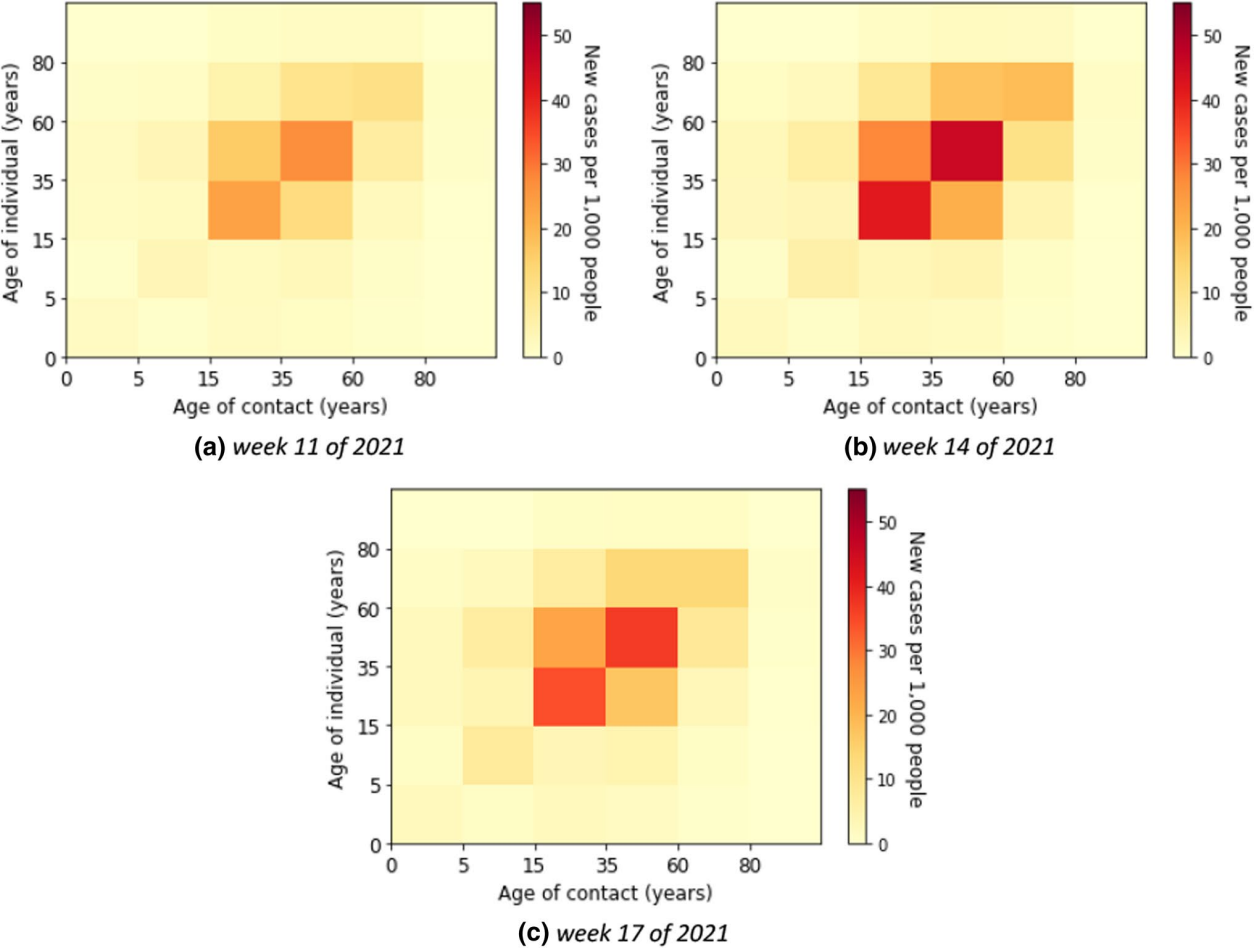
Estimated contribution of contact by age group to transmission in the third wave

The age-specific forces of infection by age group for the third wave (i.e. weeks 7 until 20 of 2021) are shown in heat maps in Fig. 9. Different from the first and second waves, the individual contribution of contacts to the transmission rates was highest in children and young adults. The absolute contribution of contacts to the transmission in the third wave shows rather similar trends as in the second wave (see Fig. 10).

**Fig. 11:**
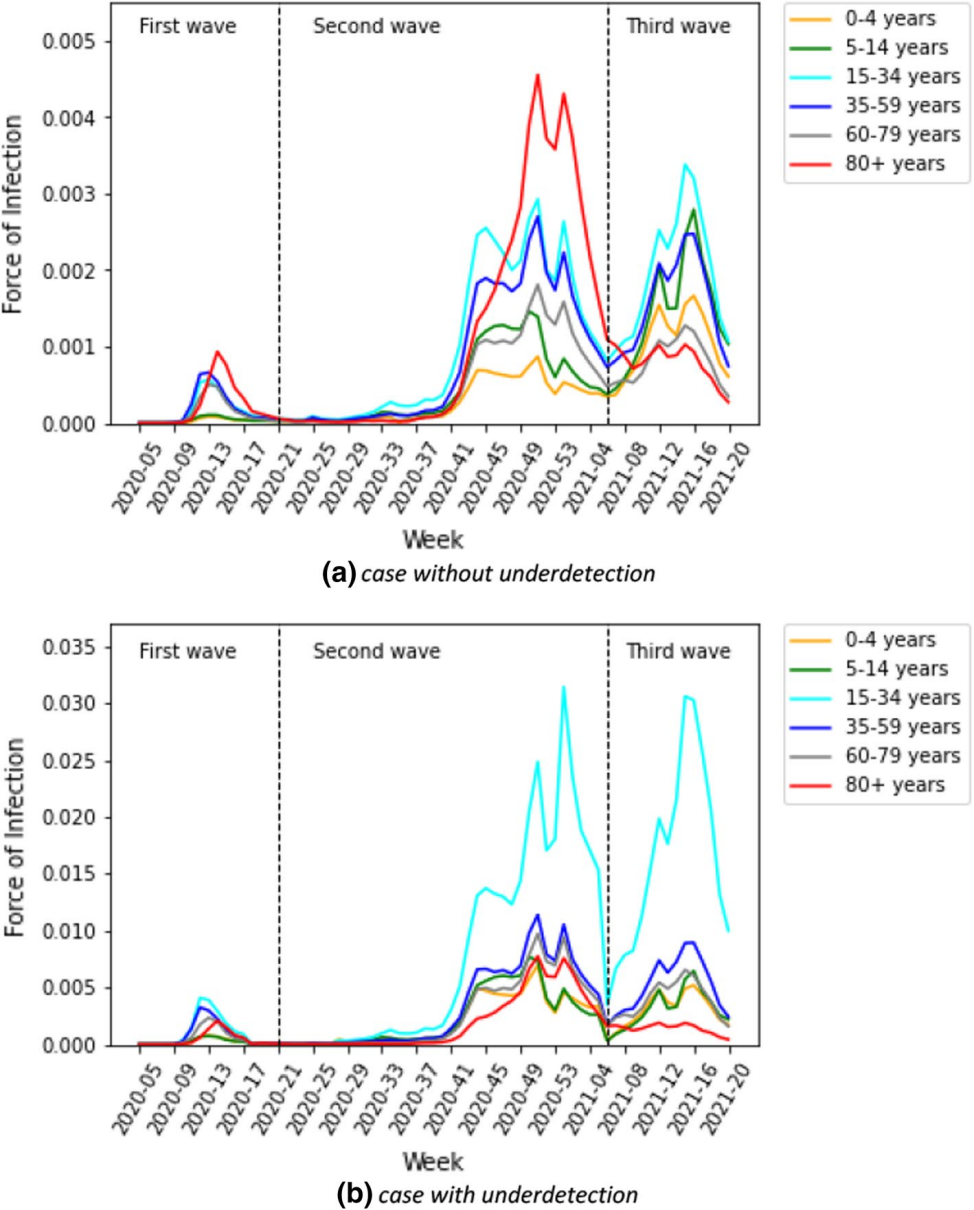
Estimated force of infection by age group for case **a** without and **b** with underdetection

Figure 11a shows the estimated force of infection by age group. In the first wave, the trends of the adult age groups appear to be homogeneous. The peak of transmission in elderly groups in the first wave appeared with a delay in comparison to the other age groups. However, they differed across adult age groups in the third wave. The force of infection in children in the third wave are higher than in the previous wave. In contrast, the trends of the elderly groups are lower in the third wave than in the previous waves.

### Contribution of age-dependent contacts to cases accounting for underdetection

The effective reproduction number taking underdetection into account is shown in Fig. 3b. The trend of ${R}_{t}$ appears to be similar to the case without underreporting. However generally, the value of ${R}_{t}$ is smaller in the underdetection analysis. At the beginning of the first wave, the value of ${R}_{t}$ for underdetection is higher. In calendar week 9 of 2020, the estimation of ${R}_{t}$, when accounting for underreporting, is 1.9 times higher. The value of ${R}_{t}$ is also higher at calendar week 28 of the second wave. At the other times, the estimation of ${R}_{t}$ ranges between 0.7 and 2.9 in the second wave and between 0.35 and 1.4 in the third wave.

**Fig. 12:**
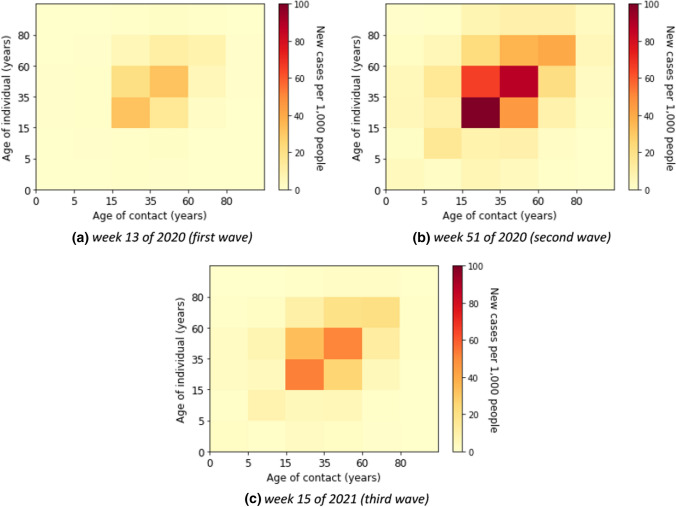
Estimated absolute contribution of contacts to the transmission accounting for underdetection

The individual forces of infection by age group are shown in heat maps in Supplementary G. The estimated absolute contributions of contacts to the transmission over time in heat maps are shown in Supplementary H. Accounting for the underdetection, Fig. 12 shows absolute contributions of contacts to the transmission at a fixed time for all waves. The figure has the same interpretation as Fig. 4, however, with different scales. We can observe that the dominant contribution of contacts to the transmission is shifted to the younger age group (young adult group), the difference due to accounting for underdetection within the model. In Fig. 11b, we show the estimated force of infection for during the first three waves if underdetection is taken into account. Here, in contrast to assessing contribution of contacts in age groups without underdetection taken into account, the estimated force of infection is much higher in the young adult group than in the other age groups in the second and third waves. In addition, the estimated force of infection in the elderly is lower in the second wave than in the case without underreporting. The delay of an increasing force of infection in older age groups is more pronounced in this simulation as apparent in Fig. 11b.

### Contribution of school setting-specific contacts to population-level cases

**Fig. 13:**
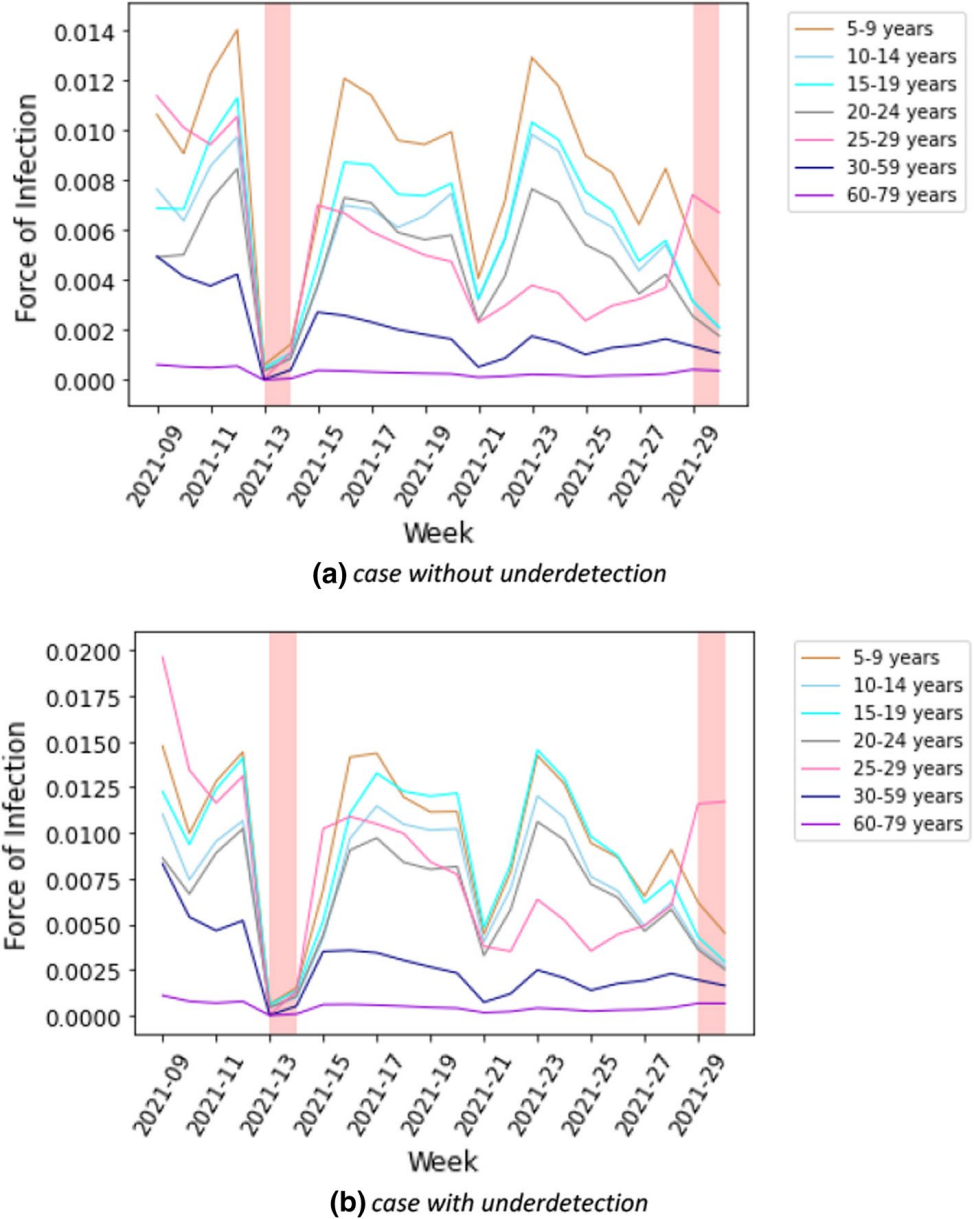
Estimated force of infection in schools by age group in the third wave for case **a** without and **b** with underdetection

**Fig. 14:**
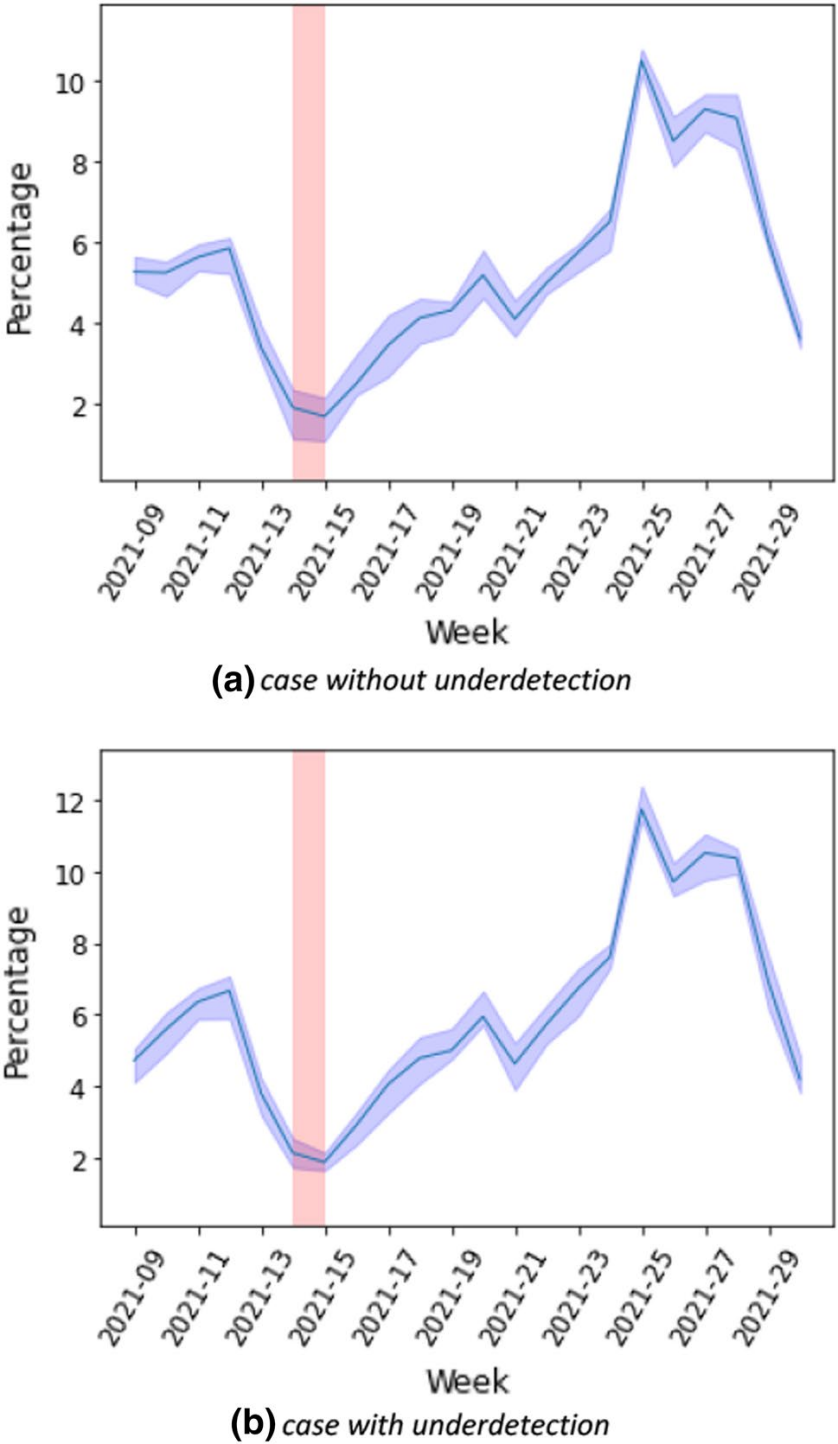
Estimated proportion of infection (CI: 95%) due to contact with infected people in schools in the third wave for case **a** without and **b** with underdetection

In the next step, we investigated the contribution of schools to the transmission of SARS-CoV-2 during the third wave in the German population. This is presented in more detail in Heinsohn et al. [19]. Using data reported for schools as well as a school contact matrix, we obtained the estimated forces of infection in schools by age group in Fig. 13a. There was a clear trough in calendar weeks 13–14 of 2021 due to the Easter holidays. Otherwise, trends appear to be very heterogeneous across age groups. We show the estimated proportion of infection due to contacts with infected people in schools in Fig. 14a. The proportion of the contribution of contacts in schools to the overall cases in the population during the third wave was up to 12%. The proportion of infections due to contact with infected people in schools declined from calendar week 25 (Fig. 14). This was due to the summer vacation which started in some German states already in week 25 while most German states were on summer vacation until week 30. Accounting for underdetection ratios in the third wave, the estimated forces of infection in schools by age group are illustrated in Fig. 13b. The estimated force of infection (after accounting for the underdetection) in the age group 5–9 years was considerably lower than when taking underdetection not into account. In contrast, the forces of infection estimated after accounting for underdetection in the age groups 25–29 years and 30–59 years were higher for than when taking underdetection into account. Figure 14b shows the proportion of the contribution of contacts in schools with underdetection ratios to the overall cases in the population.

### The Delta to Omicron SARS-CoV-2 variant transition in Germany as an example for the need for age-dependent models

To further evaluate the importance of age-dependent models, we used the constructed age-specific SEIRS model and fitted age-specific hospitalization risks for both a hypothetical Delta wave and Omicron wave in January in Germany. We estimated absolute hospitalizations for both adults and children for the next 30 weeks. We fitted the model for infections and hospitalizations through the end of January 2022 and for calendar weeks 27 to 48 of 2021 for hospitalization risks for the Delta variant.

**Fig. 15:**
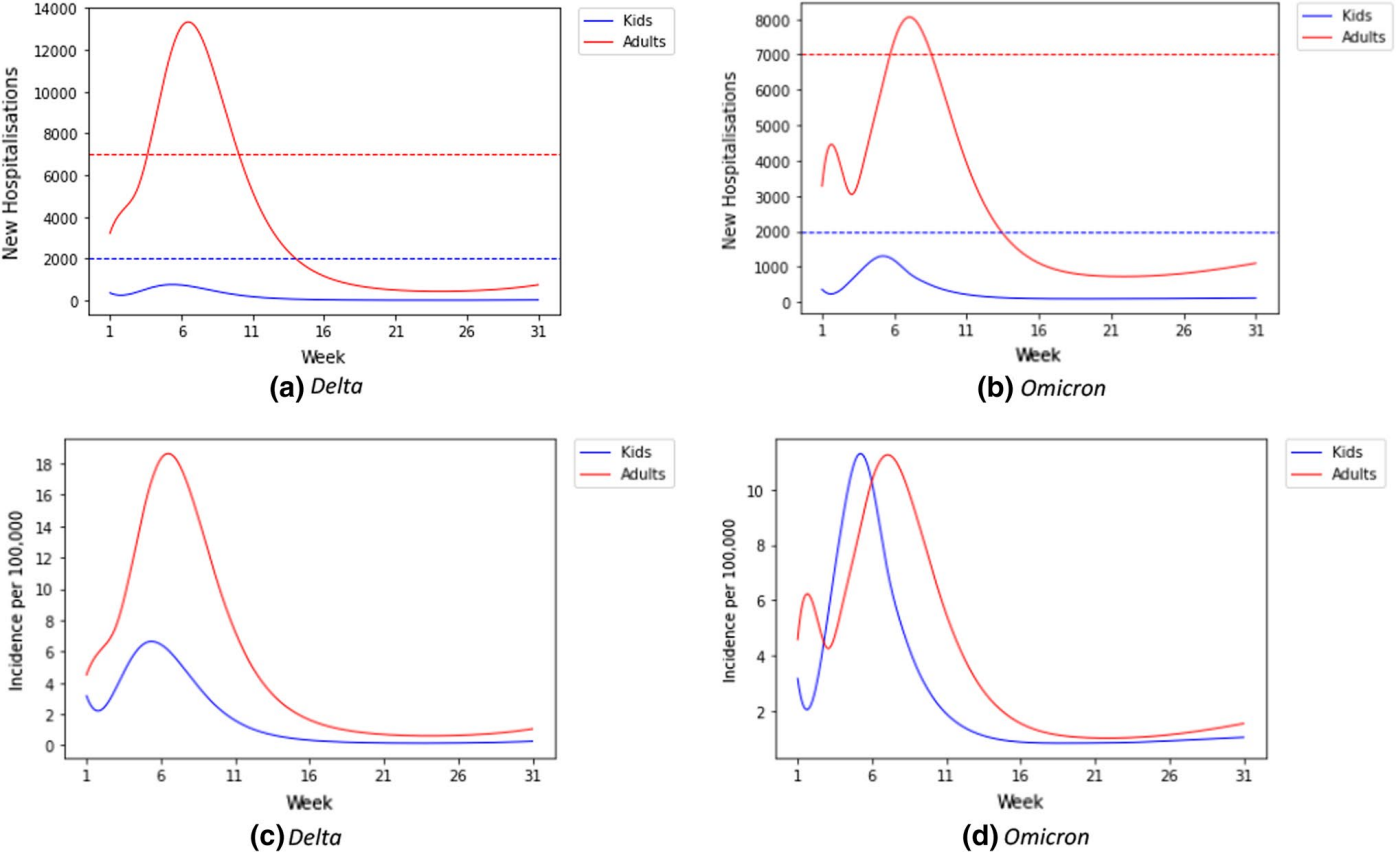
Modeling estimates the simulated hypothetical waves of new hospitalizations in adults (red) and children (blue) (**a** & **b**) and hospitalization incidences as hospitalizations/100.000 persons in adults and children (**c** & **d**) for the simulated Delta (**a** & **c**) and Omicron (**b** & **d**) waves

While most hospitalizations during the simulated wave affect adults, as in previous waves, the differences in age-specific risk reduction for Omicron as well as the overall higher incidences lead to absolute pediatric hospitalization numbers higher than previously seen (Fig. 15b and d). This also leads to a higher similarity of adult and pediatric hospitalization incidence estimates (number of hospitalizations for SARS-CoV-2 per population size) when compared to Delta modeling estimates (Fig. 15a and c). The consequence of this is that the surpassing of capacity thresholds for pediatric care could precede the one for adult care, which would be in contrast to previous waves. In supplement, we also provide the actual pediatric hospitalization in absolute as well as relative to overall hospitalizations for comparison which confirmed the trend shown in the modeling here (Supplementary A–Fig. 4 and Fig. 5).

## Discussion

We presented estimations on the course of the pandemic based on an age-specific compartmental model that accounts for differential underreporting and assesses the contribution of age- and school setting-specific contacts to overall transmission throughout the pandemic in Germany. An important part of our model is the age structure, that can lead to a better understanding of the disease spread and the decision in which groups of population interventions might be effective. The age-specific estimates are important because the healthcare system is affected differently by patients of different age groups, as specific care is required for certain age groups, and the intensity and duration of care depends on the age of the patient.

The predictive ability of our model for short-term purposes has previously been analyzed by a comparison of models contributing to a forecast to the European COVID-19 Forecast Hub [47]. In comparison to other models in Germany, shows above- average performance. For example in calendar week 14 to 38 of 2021, our model had a relative WIS 0.61 and 0.52 for weekly forecasts of cases and death. We were also able to forecast the peak of the first Omicron wave in Germany to the right week qualitatively and quantitatively within the confidence intervals of our model [47].

The age structure is one of the key determinants of SARS-CoV-2 infection patterns. Based on a systematic review and meta-analysis [46], there is evidence that individuals younger than 10 to 14 years of age have a lower susceptibility to SARS-CoV-2 infection than adults, with adolescents appearing to have a similar susceptibility to adults. Moreover, Levin et al. [25] found that the estimated age-specific infection fatality rate is very low for children and younger adults and has an exponential relationship with increasing age. Tran Kiem et al. [41] have built a modeling framework to simulate the rebound of the French epidemic in the summer of 2020, characterize age-specific transmission dynamics and evaluate different age-targeted intervention measures in the absence of vaccines. The model was proposed to evaluate the effect of shielding of older individuals on COVID-19 hospitalizations while relaxing general social distancing in the absence of vaccines.

In our example of the Delta to Omicron transition, the importance of age dependent models can be directly seen. As pediatric care facilities need to be aware of a potentially larger number of pediatric hospitalizations than in previous waves, we argue that it is important for models to provide these outputs if aiming at advising public health and political decision-making. The actual absolute hospitalizations of children during the Omicron as well as the relative share of all hospitalizations followed the trends described in the simulations presented. We were able to show that this phenomenon is easily assessable in standard modeling approaches and provides important input for healthcare practitioners as well as decision-makers. It will also be a persistent phenomenon, as children will also in the future be the group with the lowest immunization rates for SARS-CoV-2 due to newborns not being vaccinated for a yet unknown time period. We therefor see this as a relevant case for our argument that it is essential that models informing decision-making use age-specific parametrization and communicate age-specific estimates of medical resource need.

The contribution of contacts to the overall transmission across all phases of the pandemic varied but occurred predominantly in the adult group if it was not accounted for age-specific underdetection. After accounting for underreporting, however, we identified a substantial part of infections due to contacts to the young adult and teenage groups and–compared to estimates not accounting for underreporting–a higher proportion of infections in the overall population which was due to contacts in children and teens.

The difference between model estimations taking underreporting into account and those not taking underreporting into account becomes smaller towards the third wave with increased use of testing strategies targeted towards younger populations such as working age and school-age populations in Germany [1].

To understand pandemic spread in Germany, estimations assessing detailed regional spread have been provided for both the first and second waves. Lippold et al. [27] have used county-level data to provide predictions for future spread. Doblhammer et al. [12] showed that during the second wave, political affiliations and socioeconomic indicators were associated with higher incidences of SARS-CoV-2. An estimation of spreading dynamics of SARS-CoV-2 in Germany assessed regional heterogeneity in the effectiveness of measures on infection dynamics and identified three distinct regional clusters of spreading patterns [37].

We believe that the large differences in model estimations integrating age-specific underreporting and those not indicate that integrating age-specific underreporting of cases would benefit such efforts. This is in particular true, if the effectiveness of interventions or predictions are based on the contribution of certain population groups to overall transmission. Even more so, for assessments of the current epidemiological situation, these estimates should be integrated to be able to capture recent changes in epidemiological patterns. Accounting for underreporting needs to be done in an age-specific manner. Modeling without accounting for demographics likely underestimates infection activity in younger adults and relatively young populations significantly. The underestimation of the contribution of the young adult group may additionally provide a larger explanation for the overshoot of epidemic activity in Germany in the older age groups in both the second and fourth waves (Autumn 2020 and 2021). In both waves, Germany saw higher than expected epidemic activity overall starting with high epidemic activity in the younger age groups in summer. If this epidemic activity was relevantly underdetected (more than in the other age groups) this would explain the quicker than expected increase in cases and deaths in autumn as an additional explanation to the known seasonality of SARS-CoV-2. This would indicate that screening, testing, and contact tracing in this younger age group (young adults and teens) is of importance in such epidemics and pandemics even if severity of disease is not particularly high in younger age groups.

Based on information about the cases that are known to have been detected in schools, we estimated the contribution of contacts in schools to be very variable, up to 12% during calendar weeks 10 to 30 in 2021 when schools were open with hygiene measures in place. We expanded these estimations in an analysis performed on surveillance data from public health and educational agencies to the Omicron wave and found contributions of up to 20% to overall population SARS-CoV-2 cases from contacts in schools [19]. Interestingly, in contrast to what we found for age groups, this estimate was not critically affected by including underdetection estimates. This is possibly due to the mixing of age groups in this setting, lowering the underdetection effect. Also, the lack of an effect of underreporting estimates is probably caused by us not being able to include information for the third wave which in Germany had overall low underdetection and not as high age-specific underdetection differences due to testing strategies both at the workplace and in schools. Information on cases in schools is not available for previous calendar weeks. To our knowledge, no other estimations of the contribution of contacts to cases in the population from the existing data in Germany have been performed. Estimates of reduction of transmission by school closures [3, 38] for the first and second waves in Germany and Europe would indicate a larger contribution of school contacts to transmission than found by us (up to 35%); however, a part of this contribution is likely due not to cases in schools but to surrounding environments and relatives. It is also likely that the contribution of contacts in school to overall population transmission decreased in the third wave compared to previous months in Germany, as hygiene and testing measures were established within schools during that time.

In term of limitations of our approach, the value of the effective reproduction number in the first wave period may appear rather high compared to the other periods. An obvious reason for the high reproduction number in the first wave could have been caused by one or several super-spreading incidents, such as a carnival event [16]. In addition, there were artifacts due to increased testing as the first wave progressed and low case numbers. It could also be a potential overestimate, due to the rather long generation time in our model. The decrease in the effective reproduction number during the first three infection waves may indicate a reduction in contacts in the population, for example, due to the success of the non-pharmaceutical interventions. In the third wave, the force of infection in elderly groups was much lower due to the impact of vaccination and potentially higher natural infection acquisition.

The estimations made here are additionally limited by the model used attempting to capture social dynamics through an epidemic compartmental model. In particular, we scaled the contact matrix in a way that likely has an impact on the contribution of the age groups. We further only included a vaccination compartment at the end of the second wave when vaccination was recommended for the high-risk or older age group. However, the estimated force of infection does not change qualitatively by inclusion of this compartment as we have scaled the contact matrix with the scaling parameters. Another limitation is that, particularly for the third wave, we had to make assumptions regarding the underdetection in children, that are not–contrary to the other underreporting estimates–based on seroprevalence but rather on comparisons of reported cases during times with and times without large scale testing in schools.

Despite these limitations, we believed that the estimation presented here adds to the understanding of how the actual contribution of different age groups and different settings during the pandemic unfolded in Germany and could enhance both scenario and predictive modeling efforts by making the case both for using age-specific parametrization of models as well as including population-based underdetection estimates.

## Conclusions

We showed that taking underreporting into account, younger adults, and teenagers were the main contributors to infections during the first three pandemic waves in Germany. Overall, the contribution of contacts in schools to the total cases in the population was up to 12% during the third wave when schools were open with hygiene measures, but relevantly higher during the Omicron wave. We showed that including age-specific parametrization in models is essential in SARS-CoV-2 and similarly age-dependent infectious diseases to provide informative output for decision makers. Accounting for age-specific underreporting is important to correctly identify those parts of the population where quarantine, testing, vaccination and contact-reduction measures are likely to be most effective and efficient.

## Supplementary Information

Below is the link to the electronic supplementary material.

Supplementary file1 (DOCX 5693 KB)
