# Supplementary material for: Age-specific contribution of contacts to transmission of SARS-CoV-2 in Germany
Source: Eur J Epidemiol. 2023 Jan 3;38(1):39–58. doi: 10.1007/s10654-022-00938-6 (PMC9807433; doi:10.1007/s10654-022-00938-6)
Supplement: Supplementary file 1 — Supplementary file1 (DOCX 5693 KB) [file 10654_2022_938_MOESM1_ESM.docx]

# Supplementary A. Data


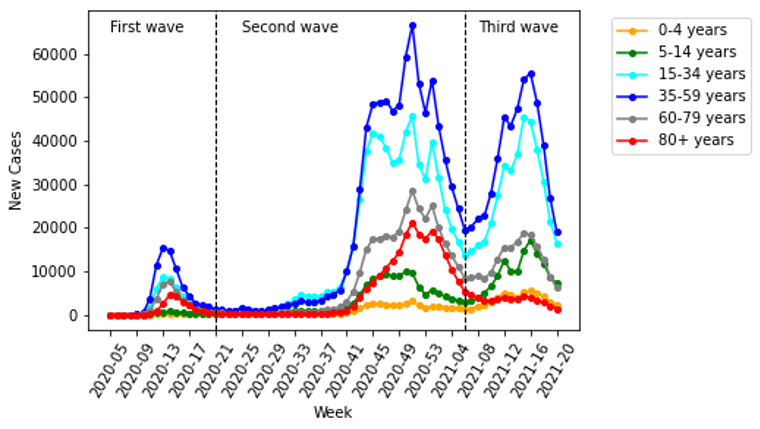


*Figure 1. The weekly reported cases for different age groups (Source: RKI [53])*


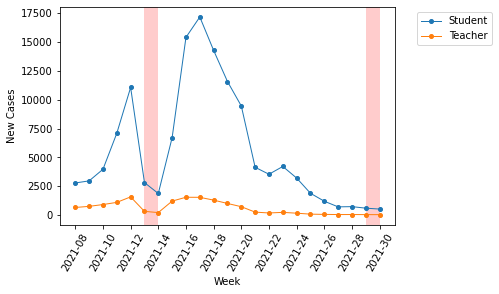


*Figure 2. The weekly reported cases for student and teacher in the third wave of 2021 (Source: KMK [55])*

Table 1. The proportion students in 2019 for different age groups (Source: BMBF [48])

| 5 – 9 years | 10 – 14 years | 15 – 19 years | 20 – 24 years |
| --- | --- | --- | --- |
| 20.47% | 28.15% | 26.51% | 17.31% |

Table 2. The proportion of teaching staff 2019/2020 for different age groups (Source: BMBF [50])

| <30 years | 30 – 39 years | 40 – 49 years | 50 – 59 years | >60 years |
| --- | --- | --- | --- | --- |
| 6.52% | 28.06% | 26.14% | 26.74% | 12.26% |


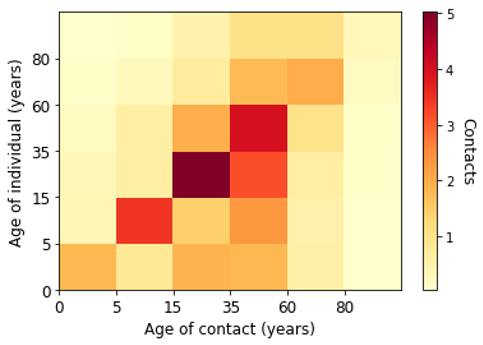


*Figure 3. Contact matrix in Germany for different age groups according to POLYMOD (Source: [32])*


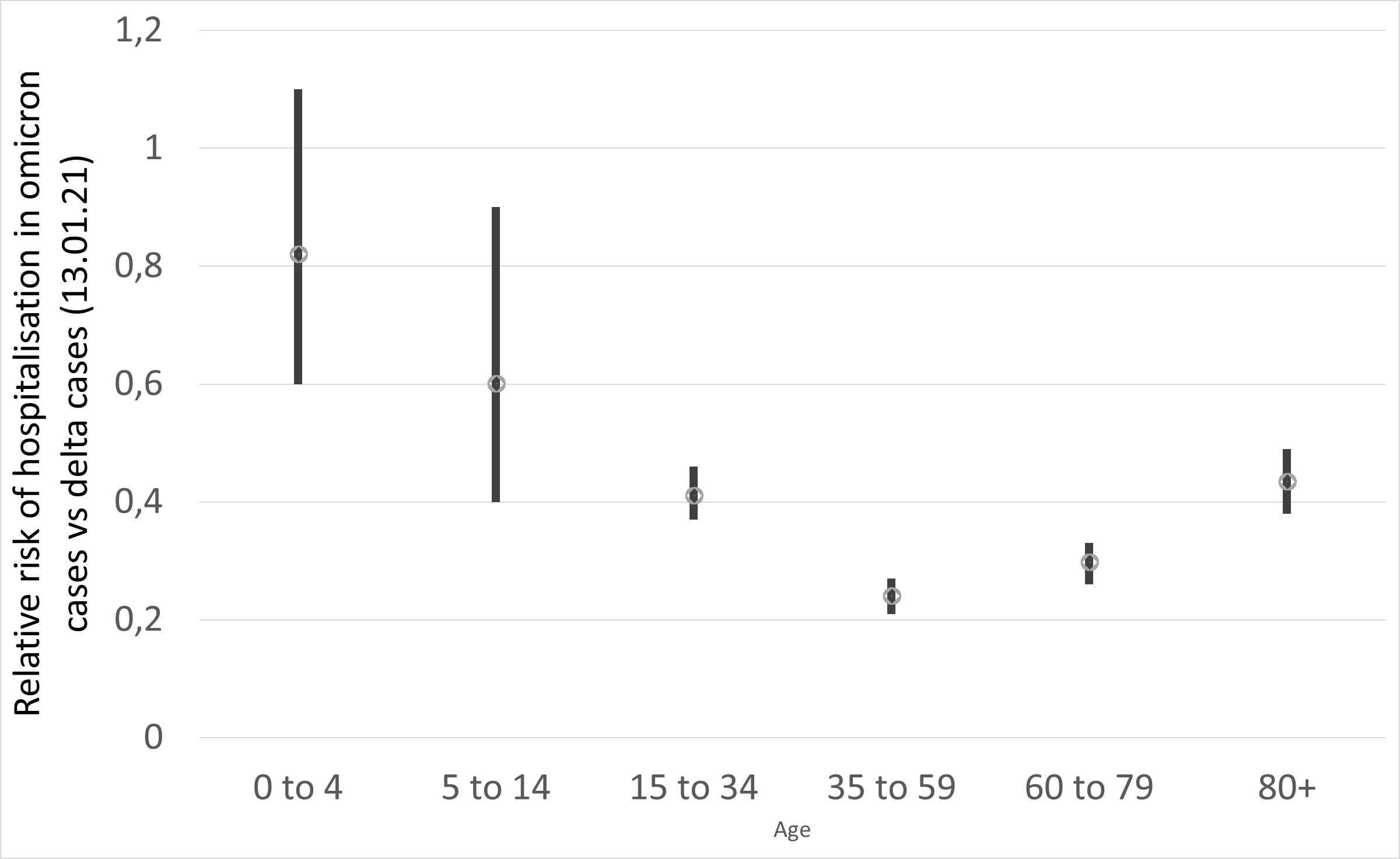


*Figure 4. Reduction in hospitalization risk for different age groups for Omicron (up to 13 January 2022) in comparison to age-specific Delta hospitalization risk in Germany based on data from the calendar weeks 27 to 48 in 2021 (Source: RKI [52])*


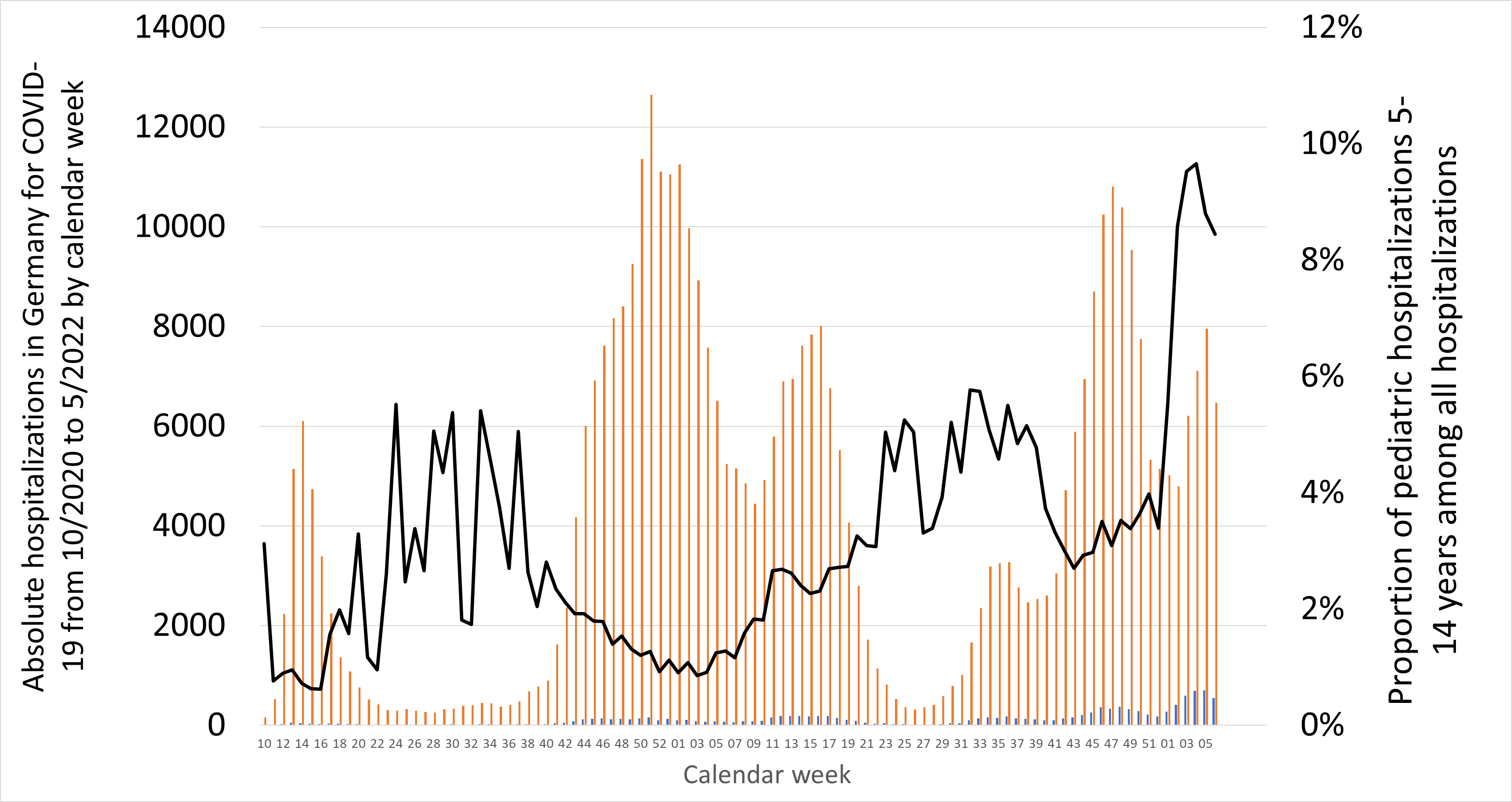


*Figure 5. Absolute number (blue, left axis) and proportion of pediatric hospitalizations(right axis, grey line) among all hospitalizations (left axis, orange) in Germany (Source: RKI [54])*

# Supplementary B. Parameters of Model

*Table 3. Parameterization by literature reviews (Source: RKI)*

| Parameter | Description |
| --- | --- |
| $P_{1}$ | The inverse of the starting infectious period (3 days) i.e. the incubation period is minus 2 days |
| $P_{2}$ | The inverse of the time a symptomatic patient recovers or requires hospitalization (4 days) |
| $P_{3}$ | The inverse of the time an asymptomatic patient recovers or dies (7 days) |
| $P_{4}$ | The inverse of the time a patient spends at a hospital before discharge (7 days) |
| $P_{5}$ | The inverse of the time span spent in ICU (10 days) |
| $P_{6}$ | The inverse of the time of a patient with long-term complication discharges (14 days) |
| $P_{7}$ | The inverse of the time span of a recovered individual with long-term complication before reinfection (90 days) |
| $P_{8}$ | The inverse of the time span of a fully recovered individual before reinfection (360 days) |
| $P_{9}$ | The inverse of the time a vaccinated individual get immunity (14 days) |
| $\varepsilon$ | The vaccination rate |
| $\beta_{s}$ | The transmission from a symptomatic infected |
| $\beta_{a}$ | The transmission from an asymptomatic infected |
| $\beta_{v}$ | The transmission from an infected after vaccination |
| $\kappa$ | The fraction of symptomatic infected |
| $\alpha$ | The proportion of the symptomatic cases requiring hospitalization |
| $\delta$ | The percentage of hospitalized patients requiring ICU |
| $\vartheta$ | The proportion of the patients in ICU who will die |
| $\nu$ | The proportion of the asymptomatic who will die |
| $\rho$ | The proportion of the symptomatic cases having a long-term complication |
| $\varphi$ | The proportion of the hospitalized patients having a long-term complication |
| $\sigma$ | The proportion of the patients in ICU having a long-term complication |
| $\eta$ | The proportion of the patients with a long-term complication requiring hospitalization |
| $\gamma$ | The proportion of the patients with a long-term complication who will die |
| $\tilde{\cdot}$ | The parameter for the vaccination compartment |

*Table 4. Parametrization by age group (Source: [29])*

| Group | Symptomatic cases requiring hospitalization | Hospitalized cases requiring ICU | Symptomatic cases having a long-term complication |
| --- | --- | --- | --- |
| 0 – 4 years | 0.01% | 5.0% | 0.01% |
| 5 – 14 years | 0.12% | 5.0% | 0.1% |
| 15 – 34 years | 3.2% | 6.3% | 10% |
| 35 – 59 years | 4.9% | 12.2% | 20% |
| 60 – 79 years | 15.2% | 30.3% | 40% |
| 80+ years | 27.3% | 70.9% | 50% |

# Supplementary C. Fitting Model


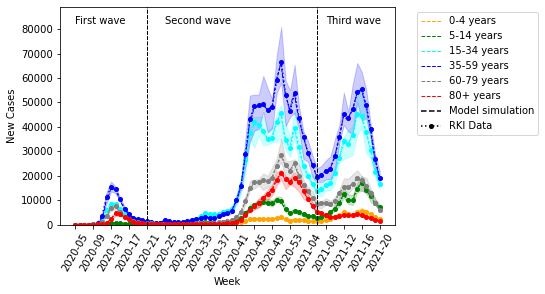


*Figure 6. Comparison between the data and the model simulation without underdetection (CI:95%)*

# Supplementary D. School-Setting Model


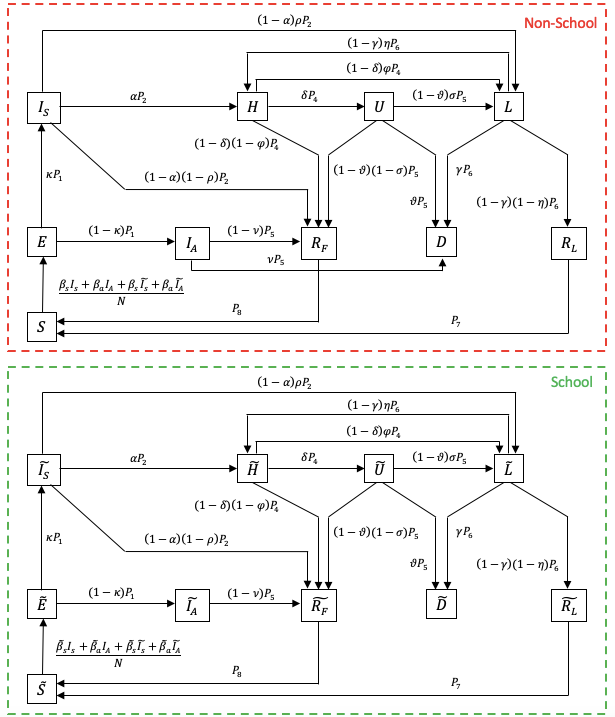


*Figure 7. Model structure for school-setting*

# Supplementary E. Estimated Marginal Force of Infection in Contacts

| *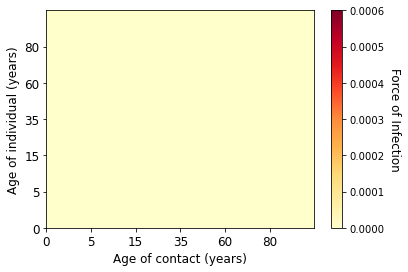*  *(a) Week 6 of 2020* | *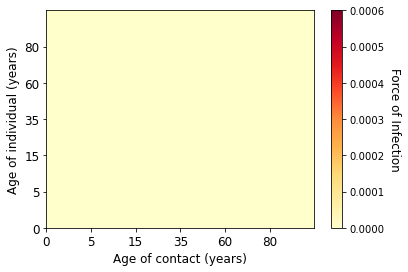*  *(b) Week 7 of 2020* |
| --- | --- |
| *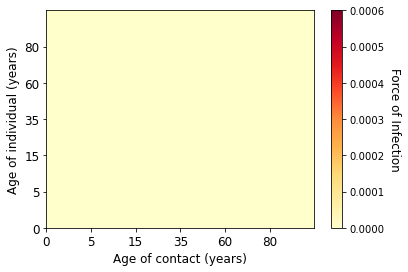*  *(c) Week 8 of 2020* | *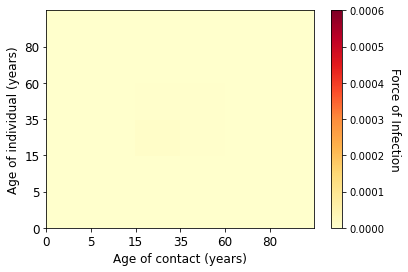*  *(d) Week 9 of 2020* |
| *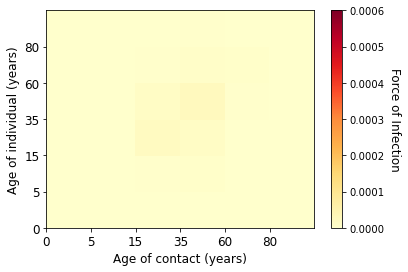*  *(e) Week 10 of 2020* | *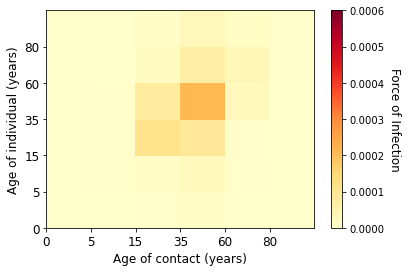*  *(f) Week 11 of 2020* |
| *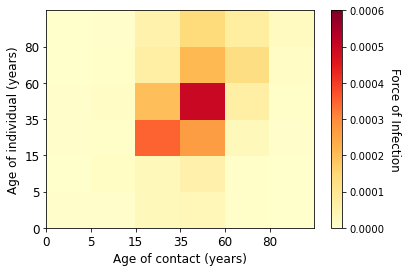*  *(g) Week 12 of 2020* | *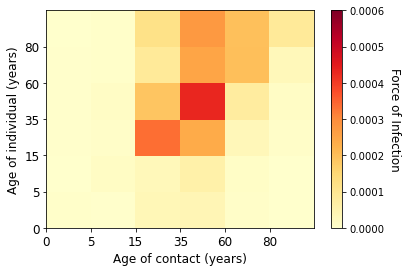*  *(h) Week 13 of 2020* |
| *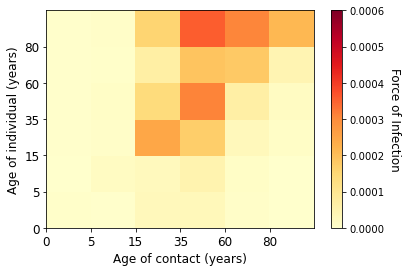*  *(i) Week 14 of 2020* | *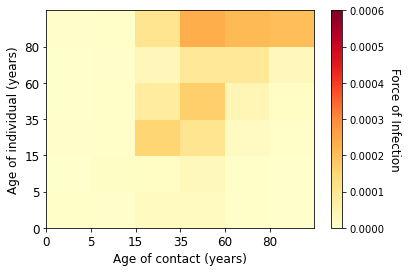*  *(j) Week 15 of 2020* |
| *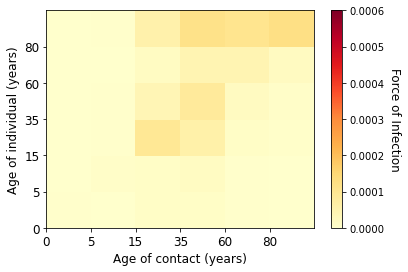*  *(k) Week 16 of 2020* | *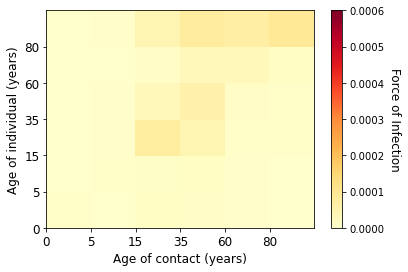*  *(l) Week 17 of 2020* |
| *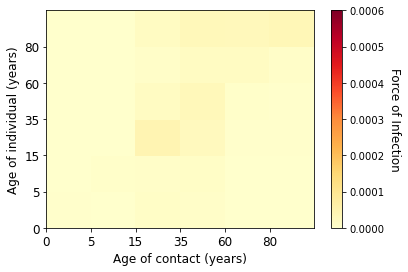*  *(m) Week 18 of 2020* | *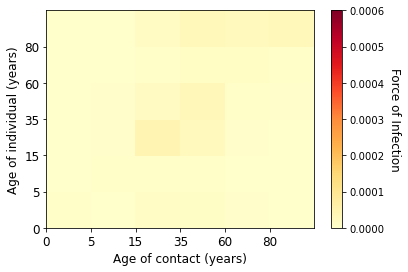*  *(n) Week 19 of 2020* |
| *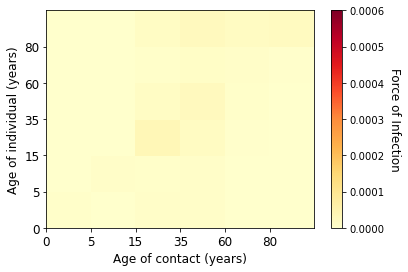*  *(o) Week 20 of 2020* | *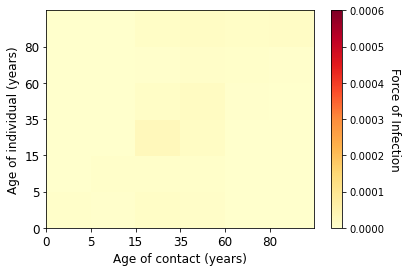*  *(p) Week 21 of 2020* |

*Figure 8. Estimated marginal force of infection for each age groups in the first wave*

| *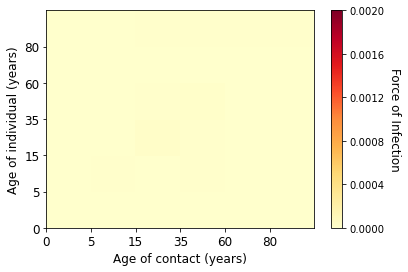*  *(a) Week 22 of 2020* | *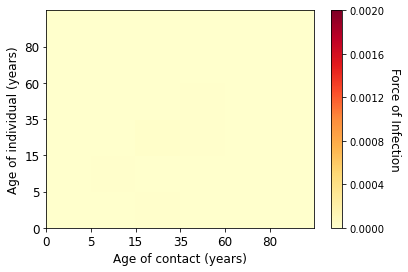*  *(b) Week 23 of 2020* |
| --- | --- |
| *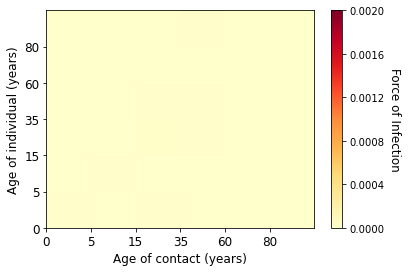*  *(c) Week 24 of 2020* | *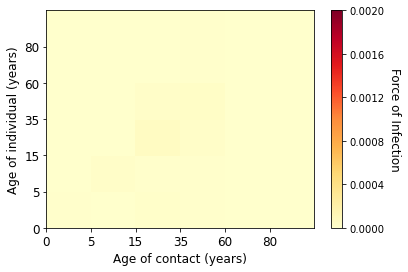*  *(d) Week 25 of 2020* |
| *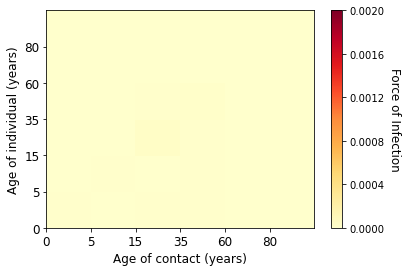*  *(e) Week 26 of 2020* | *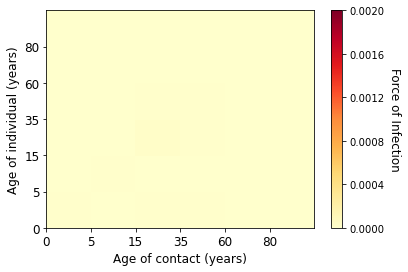*  *(f) Week 27 of 2020* |
| *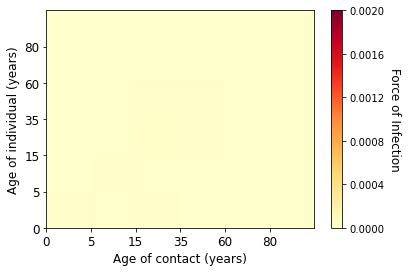*  *(g) Week 28 of 2020* | *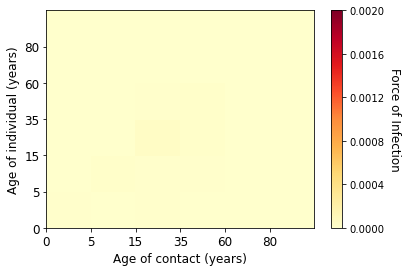*  *(h) Week 29 of 2020* |
| *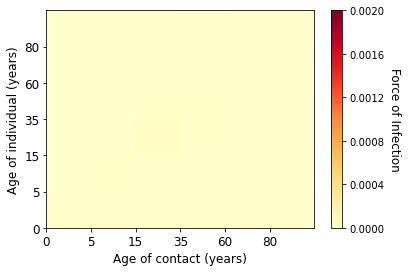*  *(i) Week 30 of 2020* | *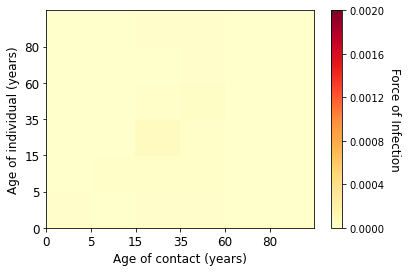*  *(j) Week 31 of 2020* |
| *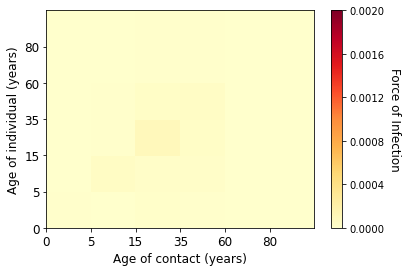*  *(k) Week 32 of 2020* | *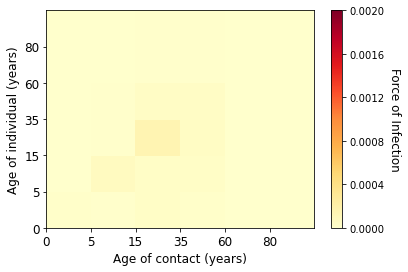*  *(l) Week 33 of 2020* |
| *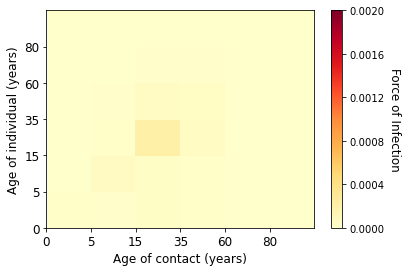*  *(m) Week 34 of 2020* | *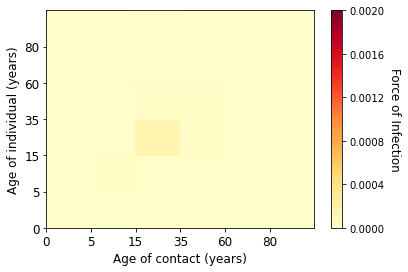*  *(n) Week 35 of 2020* |
| *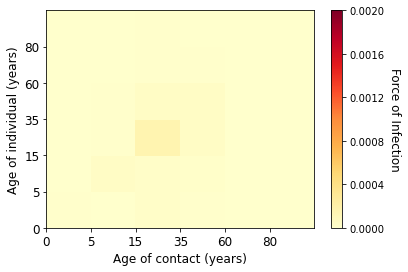*  *(o) Week 36 of 2020* | *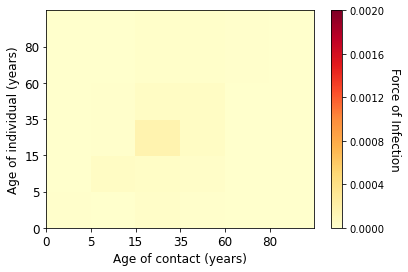*  *(p) Week 37 of 2020* |
| *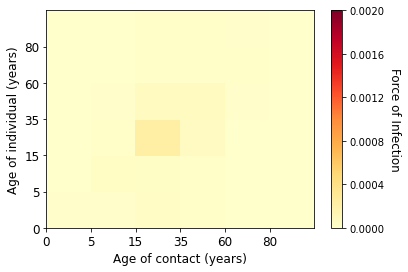*  *(q) Week 38 of 2020* | *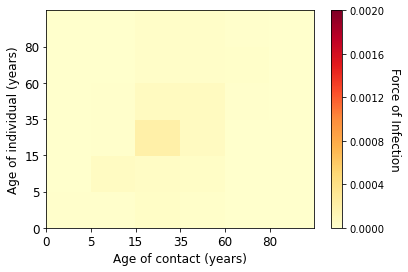*  *(r) Week 39 of 2020* |
| *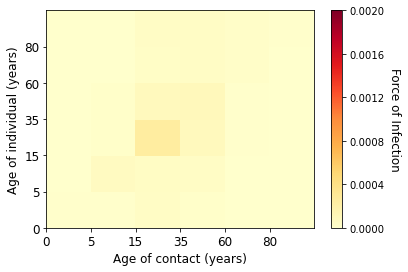*  *(s) Week 40 of 2020* | *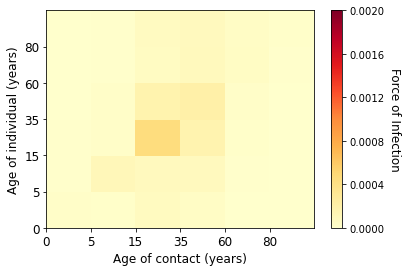*  *(t) Week 41 of 2020* |
| *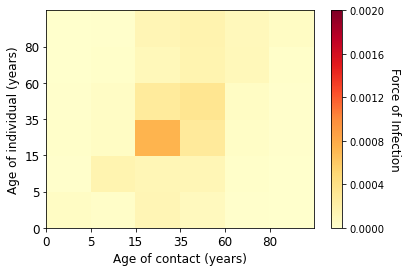*  *(u) Week 42 of 2020* | *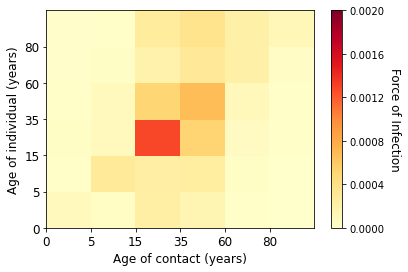*  *(v) Week 43 of 2020* |
| *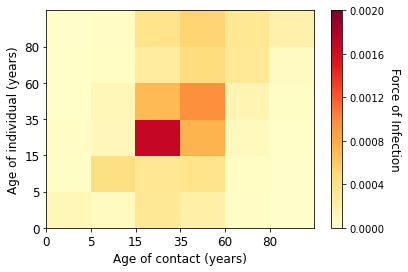*  *(w) Week 44 of 2020* | *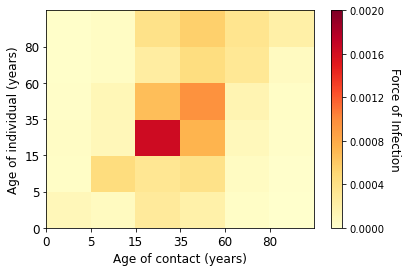*  *(x) Week 45 of 2020* |
| *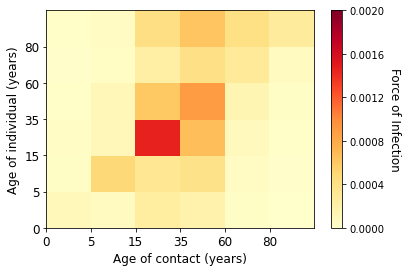*  *(y) Week 46 of 2020* | *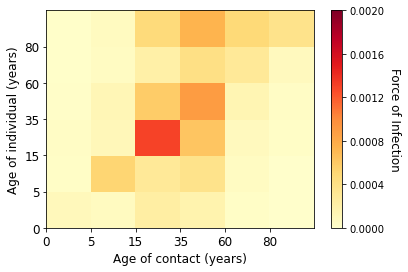*  *(z) Week 47 of 2020* |
| *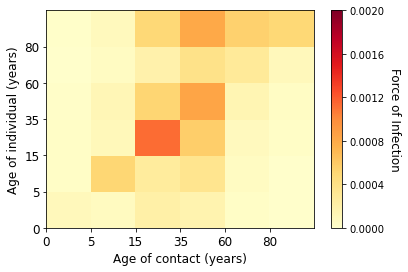*  *(aa) Week 48 of 2020* | *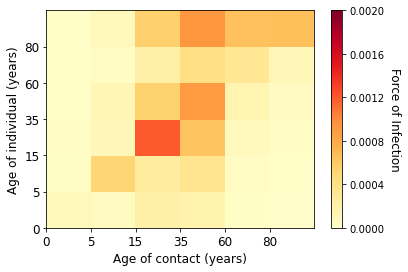*  *(ab) Week 49 of 2020* |
| *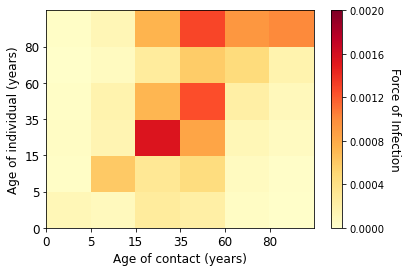*  *(ac) Week 50 of 2020* | *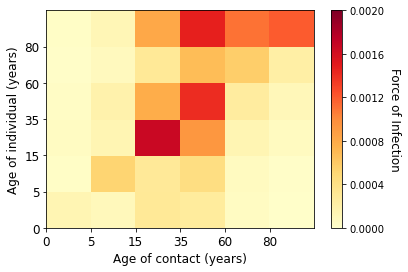*  *(ad) Week 51 of 2020* |
| *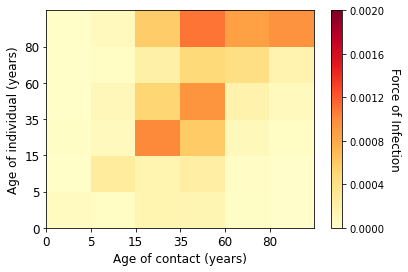*  *(ae) Week 52 of 2020* | *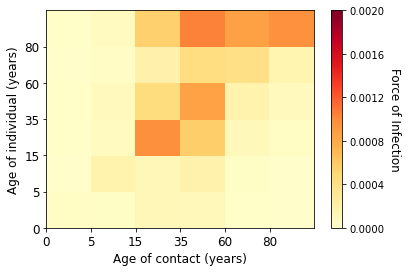*  *(af) Week 53 of 2020* |
| *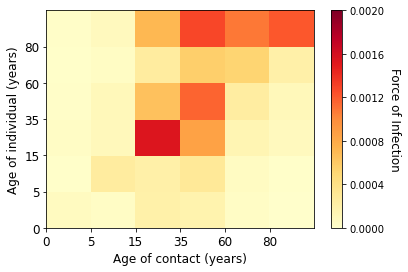*  *(ag) Week 1 of 2021* | *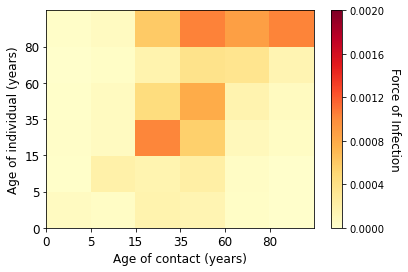*  *(ah) Week 2 of 2021* |
| *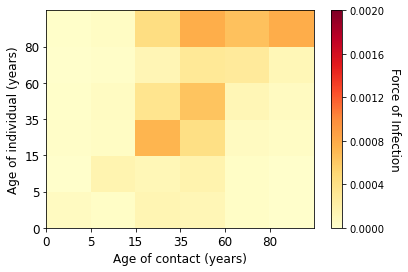*  *(ai) Week 3 of 2021* | *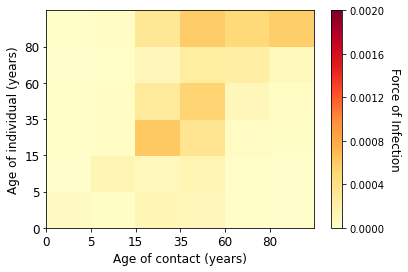*  *(aj) Week 4 of 2021* |
| *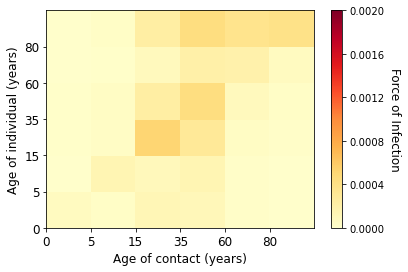*  *(ak) Week 5 of 2021* | *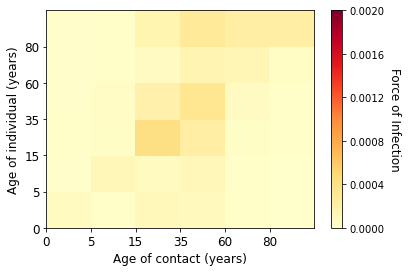*  *(al) Week 6 of 2021* |

*Figure 9. Estimated marginal force of infection for each age groups in the second wave*

| *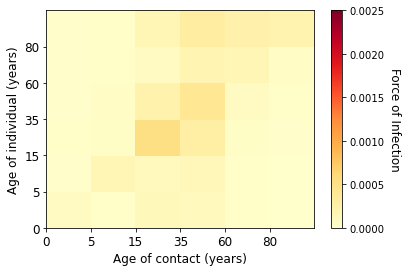*  *(a) Week 7 of 2021* | *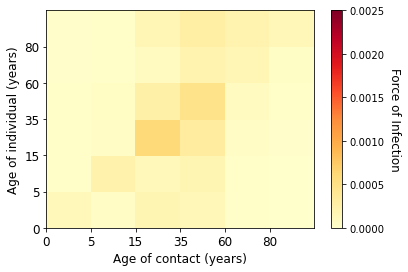*  *(b) Week 8 of 2021* |
| --- | --- |
| *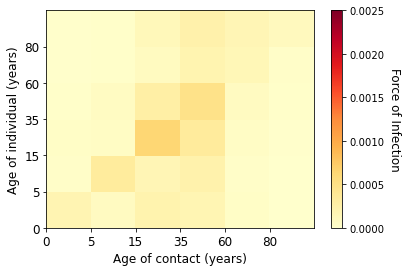*  *(c) Week 9 of 2021* | *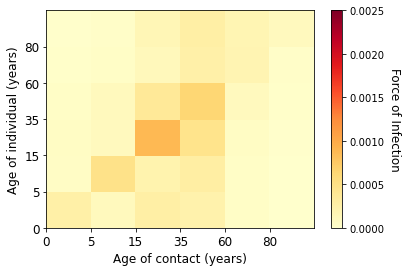*  *(d) Week 10 of 2021* |
| *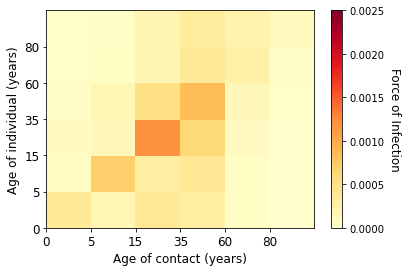*  *(e) Week 11 of 2021* | *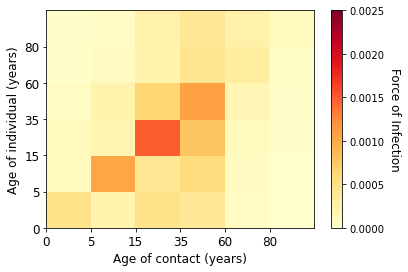*  *(f) Week 12 of 2021* |
| *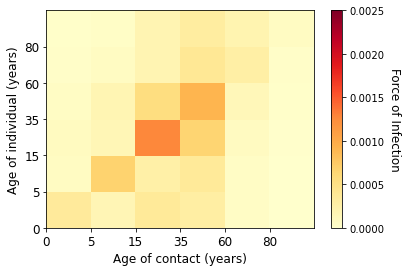*  *(g) Week 13 of 2021* | *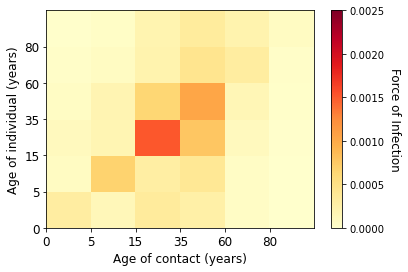*  *(h) Week 14 of 2021* |
| *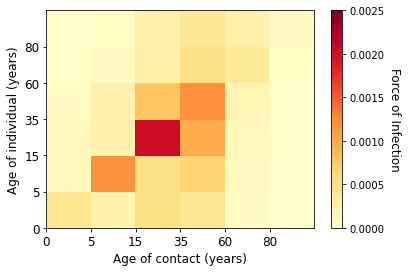*  *(i) Week 15 of 2021* | *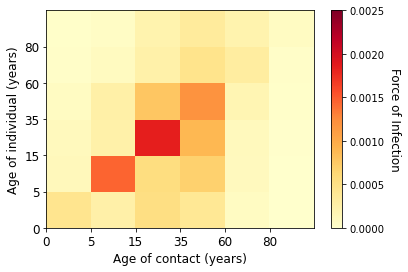*  *(j) Week 16 of 2021* |
| *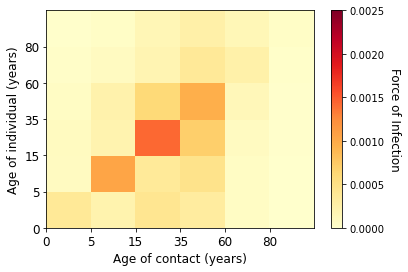*  *(k) Week 17 of 2021* | *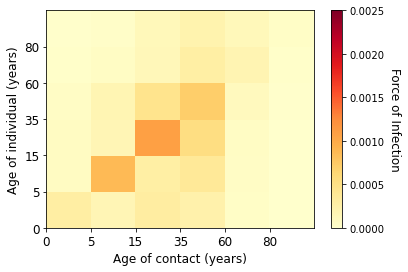*  *(l) Week 18 of 2021* |
| *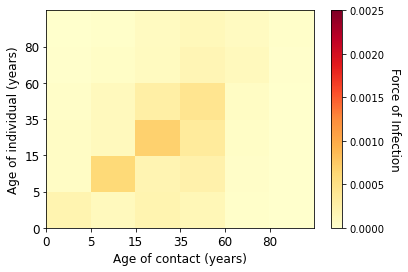*  *(m) Week 19 of 2021* | *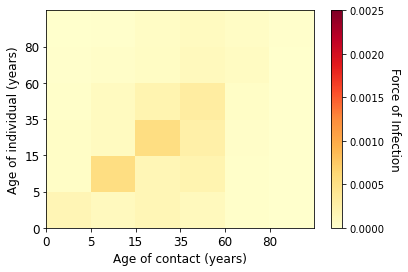*  *(n) Week 20 of 2021* |

*Figure 10. Estimated marginal force of infection for each age groups in the third wave*

# Supplementary F. Estimated Contribution of Contacts to Transmission

| *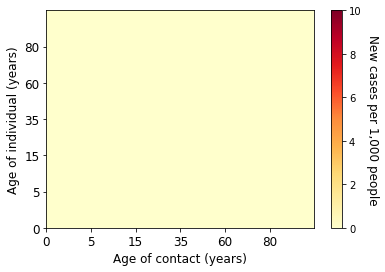*  *(a) Week 6 of 2020* | *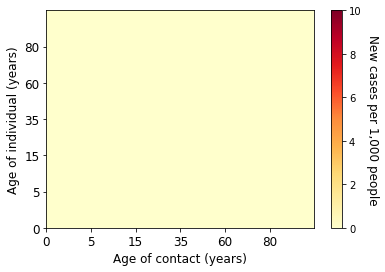*  *(b) Week 7 of 2020* |
| --- | --- |
| *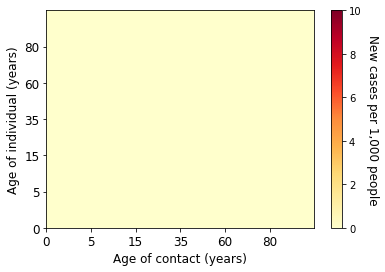*  *(c) Week 8 of 2020* | *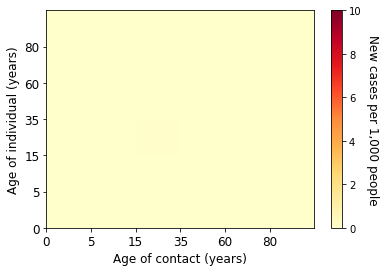*  *(d) Week 9 of 2020* |
| *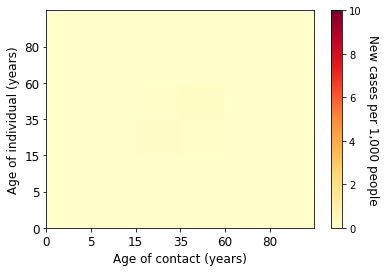*  *(e) Week 10 of 2020* | *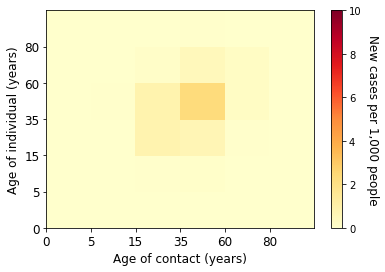*  *(f) Week 11 of 2020* |
| *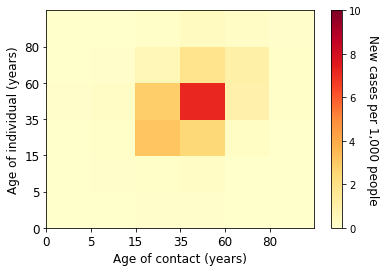*  *(g) Week 12 of 2020* | *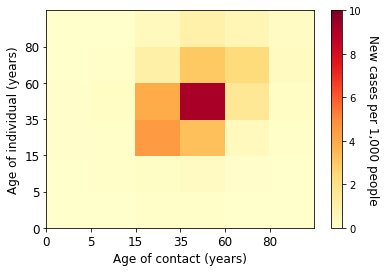*  *(h) Week 13 of 2020* |
| *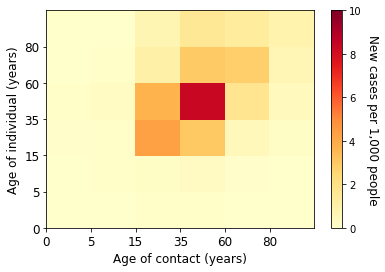*  *(i) Week 14 of 2020* | *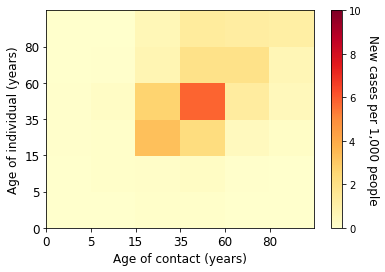*  *(j) Week 15 of 2020* |
| *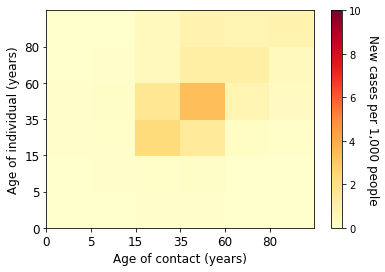*  *(k) Week 16 of 2020* | *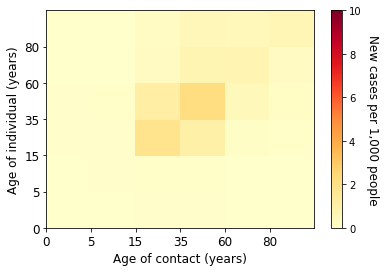*  *(l) Week 17 of 2020* |
| *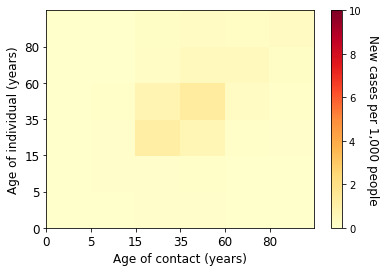*  *(m) Week 18 of 2020* | *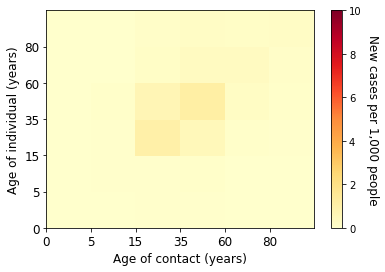*  *(n) Week 19 of 2020* |
| *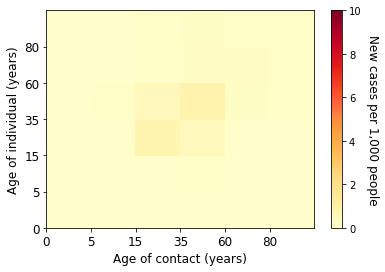*  *(o) Week 20 of 2020* | *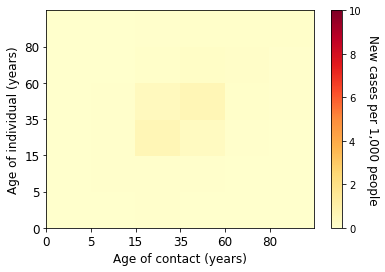*  *(p) Week 21 of 2020* |

*Figure 11. Estimated absolute contribution of transmission for each age groups in the first wave*

| *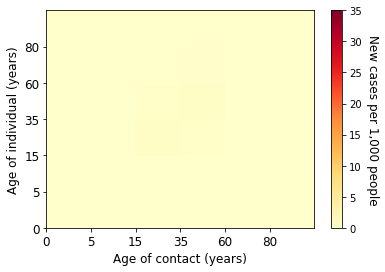*  *(a) Week 22 of 2020* | *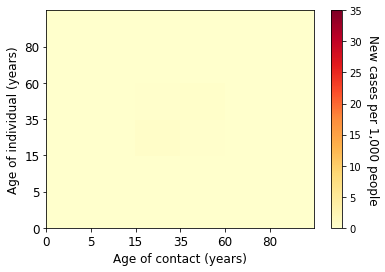(b) Week 23 of 2020* |
| --- | --- |
| *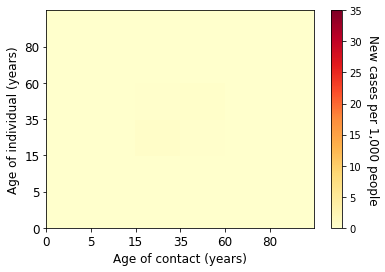*  *(c) Week 24 of 2020* | *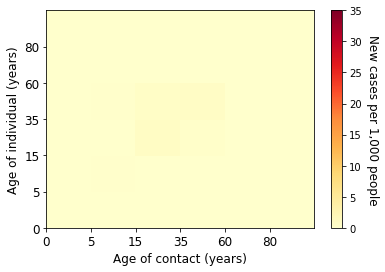*  *(d) Week 25 of 2020* |
| *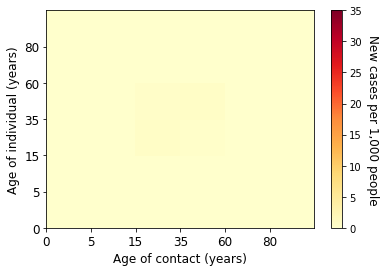*  *(e) Week 26 of 2020* | *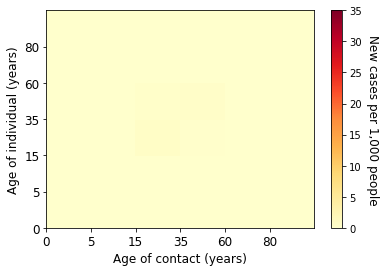*  *(f) Week 27 of 2020* |
| *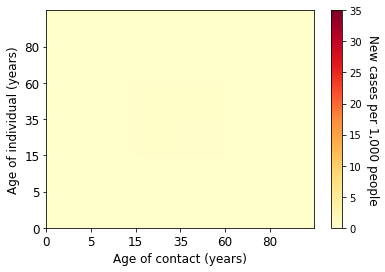*  *(g) Week 28 of 2020* | *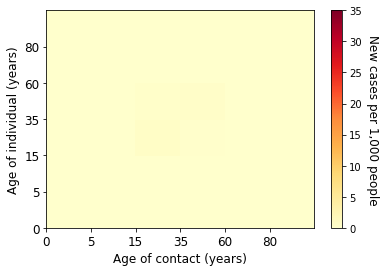*  *(h) Week 29 of 2020* |
| *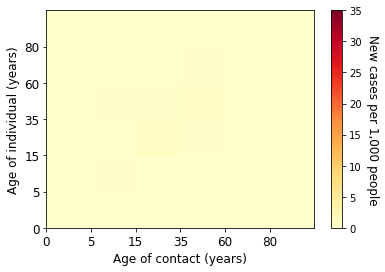*  *(i) Week 30 of 2020* | *(j) Week 31 of 2020* |
| *(k) Week 32 of 2020* | *(l) Week 33 of 2020* |
| *(m) Week 34 of 2020* | *(n) Week 35 of 2020* |
| *(o) Week 36 of 2020* | *(p) Week 37 of 2020* |
| *(q) Week 38 of 2020* | *(r) Week 39 of 2020* |
| *(s) Week 40 of 2020* | *(t) Week 41 of 2020* |
| *(u) Week 42 of 2020* | *(v) Week 43 of 2020* |
| *(w) Week 44 of 2020* | *(x) Week 45 of 2020* |
| *(y) Week 46 of 2020* | *(z) Week 47 of 2020* |
| *(aa) Week 48 of 2020* | *(ab) Week 49 of 2020* |
| *(ac) Week 50 of 2020* | *(ad) Week 51 of 2020* |
| *(ae) Week 52 of 2020* | *(af) Week 53 of 2020* |
| *(ag) Week 1 of 2021* | *(ah) Week 2 of 2021* |
| *(ai) Week 3 of 2021* | *(aj) Week 4 of 2021* |
| *(ak) Week 5 of 2021* | *(al) Week 6 of 2021* |

*Figure 12. Estimated absolute contribution of transmission for each age groups in the second wave*

| *(a) Week 7 of 2021* | *(b) Week 8 of 2021* |
| --- | --- |
| *(c) Week 9 of 2021* | *(d) Week 10 of 2021* |
| *(e) Week 11 of 2021* | *(f) Week 12 of 2021* |
| *(g) Week 13 of 2021* | *(h) Week 14 of 2021* |
| *(i) Week 15 of 2021* | *(j) Week 16 of 2021* |
| *(k) Week 17 of 2021* | *(l) Week 18 of 2021* |
| *(m) Week 19 of 2021* | *(n) Week 20 of 2021* |

*Figure 13. Estimated absolute contribution of transmission for each age groups in the third wave*

# Supplementary H. Estimated Marginal Force of Infection in Contacts with the Underdetection Ratios

| *(a) Week 6 of 2020* | *(b) Week 7 of 2020* |
| --- | --- |
| *(c) Week 8 of 2020* | *(d) Week 9 of 2020* |
| *(e) Week 10 of 2020* | *(f) Week 11 of 2020* |
| *(g) Week 12 of 2020* | *(h) Week 13 of 2020* |
| *(i) Week 14 of 2020* | *(j) Week 15 of 2020* |
| *(k) Week 16 of 2020* | *(l) Week 17 of 2020* |
| *(m) Week 18 of 2020* | *(n) Week 19 of 2020* |
| *(o) Week 20 of 2020* | *(p) Week 21 of 2020* |

*Figure 14. Estimated marginal force of infection for each age groups in the first wave*

| *(a) Week 22 of 2020* | *(b) Week 23 of 2020* |
| --- | --- |
| *(c) Week 24 of 2020* | *(d) Week 25 of 2020* |
| *(e) Week 26 of 2020* | *(f) Week 27 of 2020* |
| *(g) Week 28 of 2020* | *(h) Week 29 of 2020* |
| *(i) Week 30 of 2020* | *(j) Week 31 of 2020* |
| *(k) Week 32 of 2020* | *(l) Week 33 of 2020* |
| *(m) Week 34 of 2020* | *(n) Week 35 of 2020* |
| *(o) Week 36 of 2020* | *(p) Week 37 of 2020* |
| *(q) Week 38 of 2020* | *(r) Week 39 of 2020* |
| *(s) Week 40 of 2020* | *(t) Week 41 of 2020* |
| *(u) Week 42 of 2020* | *(v) Week 43 of 2020* |
| *(w) Week 44 of 2020* | *(x) Week 45 of 2020* |
| *(y) Week 46 of 2020* | *(z) Week 47 of 2020* |
| *(aa) Week 48 of 2020* | *(ab) Week 49 of 2020* |
| *(ac) Week 50 of 2020* | *(ad) Week 51 of 2020* |
| *(ae) Week 52 of 2020* | *(af) Week 53 of 2020* |
| *(ag) Week 1 of 2021* | *(ah) Week 2 of 2021* |
| *(ai) Week 3 of 2021* | *(aj) Week 4 of 2021* |
| *(ak) Week 5 of 2021* | *(al) Week 6 of 2021* |

*Figure 15. Estimated marginal force of infection for each age groups in the second wave*

| *(a) Week 7 of 2021* | *(b) Week 8 of 2021* |
| --- | --- |
| *(c) Week 9 of 2021* | *(d) Week 10 of 2021* |
| *(e) Week 11 of 2021* | *(f) Week 12 of 2021* |
| *(g) Week 13 of 2021* | *(h) Week 14 of 2021* |
| *(i) Week 15 of 2021* | *(j) Week 16 of 2021* |
| *(k) Week 17 of 2021* | *(l) Week 18 of 2021* |
| *(m) Week 19 of 2021* | *(n) Week 20 of 2021* |

*Figure 16. Estimated marginal fore of infection for each age groups in the third wave*

# Supplementary I. Estimated Contribution of Contact to Transmission with the Underdetection Ratios

| *(a) Week 6 of 2020* | *(b) Week 7 of 2020* |
| --- | --- |
| *(c) Week 8 of 2020* | *(d) Week 9 of 2020* |
| *(e) Week 10 of 2020* | *(f) Week 11 of 2020* |
| *(g) Week 12 of 2020* | *(h) Week 13 of 2020* |
| *(i) Week 14 of 2020* | *(j) Week 15 of 2020* |
| *(k) Week 16 of 2020* | *(l) Week 17 of 2020* |
| *(m) Week 18 of 2020* | *(n) Week 19 of 2020* |
| *(o) Week 20 of 2020* | *(p) Week 21 of 2020* |

*Figure 17. Estimated absolute contribution of transmission for each age groups in the first wave*

| *(a) Week 22 of 2020* | *(b) Week 23 of 2020* |
| --- | --- |
| *(c) Week 24 of 2020* | *(d) Week 25 of 2020* |
| *(e) Week 26 of 2020* | *(f) Week 27 of 2020* |
| *(g) Week 28 of 2020* | *(h) Week 29 of 2020* |
| *(i) Week 30 of 2020* | *(j) Week 31 of 2020* |
| *(k) Week 32 of 2020* | *(l) Week 33 of 2020* |
| *(m) Week 34 of 2020* | *(n) Week 35 of 2020* |
| *(o) Week 36 of 2020* | *(p) Week 37 of 2020* |
| *(q) Week 38 of 2020* | *(r) Week 39 of 2020* |
| *(s) Week 40 of 2020* | *(t) Week 41 of 2020* |
| *(u) Week 42 of 2020* | *(v) Week 43 of 2020* |
| *(w) Week 44 of 2020* | *(x) Week 45 of 2020* |
| *(y) Week 46 of 2020* | *(z) Week 47 of 2020* |
| *(aa) Week 48 of 2020* | *(ab) Week 49 of 2020* |
| *(ac) Week 50 of 2020* | *(ad) Week 51 of 2020* |
| *(ae) Week 52 of 2020* | *(af) Week 53 of 2020* |
| *(ag) Week 1 of 2021* | *(ah) Week 2 of 2021* |
| *(ai) Week 3 of 2021* | *(aj) Week 4 of 2021* |
| *(ak) Week 5 of 2021* | *(al) Week 6 of 2021* |

*Figure 18. Estimated absolute contribution of transmission for each age groups in the second wave*

| *(a) Week 7 of 2021* | *(b) Week 8 of 2021* |
| --- | --- |
| *(c) Week 9 of 2021* | *(d) Week 10 of 2021* |
| *(e) Week 11 of 2021* | *(f) Week 12 of 2021* |
| *(g) Week 13 of 2021* | *(h) Week 14 of 2021* |
| *(i) Week 15 of 2021* | *(j) Week 16 of 2021* |
| *(k) Week 17 of 2021* | *(l) Week 18 of 2021* |
| *(m) Week 19 of 2021* | *(n) Week 20 of 2021* |

*Figure 19. Estimated absolute contribution of transmission for each age groups in the third wave*
